# Correlating Valence and 2p3d RIXS Spectroscopies: A Ligand-Field Study of Spin-Crossover Iron(II)

**DOI:** 10.1021/acs.inorgchem.4c00435

**Published:** 2024-04-08

**Authors:** Casey Van Stappen, Benjamin E. Van Kuiken, Max Mörtel, Kari O. Ruotsalainen, Dimitrios Maganas, Marat M. Khusniyarov, Serena DeBeer

**Affiliations:** † Max Planck Institute for Chemical Energy Conversion, Stiftstrasse 34-36, 45470 Mülheim an der Ruhr, Germany; ‡ European XFEL, Holzkoppel 4, 22869 Schenefeld, Germany; § Department of Chemistry and Pharmacy, Friedrich-Alexander-Universität Erlangen-Nürnberg (FAU), Egerlandstrasse 1, 91058 Erlangen, Germany; ∥ Synchrotron SOLEIL, L‘Orme des Merisiers, Départementale 128, 91190 Saint-Aubin, France; ⊥ Max-Planck-Institut für Kohlenforschung, Kaiser-Wilhelm-Platz 1, 45470 Mülheim an der Ruhr, Germany

## Abstract

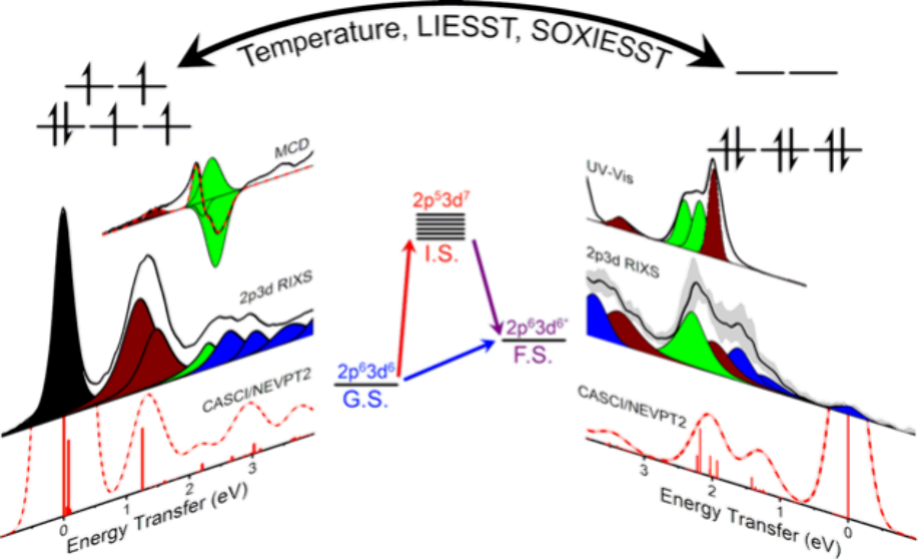

The molecular spin-crossover phenomenon between high-spin (HS) and low-spin (LS) states is a promising route to next-generation information storage, sensing applications, and molecular spintronics. Spin-crossover complexes also provide a unique opportunity to study the ligand field (LF) properties of a system in both HS and LS states while maintaining the same ligand environment. Presently, we employ complementing valence and core-level spectroscopic methods to probe the electronic excited-state manifolds of the spin-crossover complex [Fe^II^(H_2_B(pz)_2_)_2_phen]^0^. Light-induced excited spin-state trapping (LIESST) at liquid He temperatures is exploited to characterize magnetic and spectroscopic properties of the photoinduced HS state using SQUID magnetometry and magnetic circular dichroism spectroscopy. In parallel, Fe 2p3d RIXS spectroscopy is employed to examine the Δ*S* = 0, 1 excited LF states. These experimental studies are combined with state-of-the-art CASSCF/NEVPT2 and CASCI/NEVPT2 calculations characterizing the ground and LF excited states. Analysis of the acquired LF information further supports the notion that the spin-crossover of [Fe^II^(H_2_B(pz)_2_)_2_phen]^0^ is asymmetric, evidenced by a decrease in e_π_ in the LS state. The results demonstrate the power of cross-correlating spectroscopic techniques with high and low LF information content to make accurate excited-state assignments, as well as the current capabilities of ab initio theory in interpreting these electronic properties.

## Abstract 2

Valence and 2p3d RIXS spectroscopies are combined with the CASCI/NEVPT2 level of theory to investigate the temperature-dependent changes of the ligand field environment in an Fe^II^ spin-crossover complex.

## Introduction

Molecular spin-crossover (SCO) compounds are bistable molecules that display reversible switching between high-spin (HS) and low-spin (LS) electronic states as a function of an external perturbation such as temperature, light, pressure, magnetic field, or electrical and chemical stimuli.^1−5^ These complexes continue to be the subject of intensive studies due to their prospective uses in high-density molecule-based information storage, display devices, photovoltaics, sensing applications, and molecular electronics and spintronics.^4−9^ In order to understand how these systems function and further rationally modulate their properties, an intimate understanding of their electronic structure is critical. Importantly, the excited-state intermediate ligand-field (LF) and metal-to-ligand charge transfer (MLCT) triplet states of these systems have been found to be critical as intermediate states facilitate rapid SCO transitions.^10−12^ Therefore, approaches to better understand the excited-state manifold, including both determining Δ*S* = 1 electronic states and discriminating between different states, are necessary.

Accessing the LF states of transition-metal complexes is generally challenging for several reasons. The parity forbidden nature of d-d type transitions^13−16^ makes these features generally extremely weak when observed by traditional methods, such as UV–vis/nIR spectroscopy, when compared to charge-transfer or ligand-based transitions. The use of magnetic circular dichroism (MCD) partially relieves this problem in systems with low-symmetry paramagnetic metal centers, where intensity is imparted via spin and orbital angular momentum mechanisms.^17−20^ However, higher lying (>2 eV) LF transitions in MCD are still easily obscured by the presence of charge-transfer transitions between the metal and ligands, and lower lying states (<0.6 eV) are challenging to access instrumentally.

An alternative method to gain valence excited-state information is resonant inelastic X-ray scattering at the metal L-edge (2p3d RIXS). This technique is a core-level photon-in/photon-out spectroscopic technique that can be used to probe electronic states in the same energetic range as UV–vis/nIR/IR spectroscopies and can be conceptualized as a two-step excitation/relaxation process.^21,22^ The first step involves the excitation of a core 2p electron to the 3d shell, moving from the 2p^6^3d^*n*^ ground state to excited states involving 2p^5^3d^*n*+1^ electronic configurations. This is followed by subsequent relaxation via photon emission to fill the 2p core-hole, moving from 2p^5^3d^*n*+1^ to a manifold of 2p^6^3d^n*^ final states. This 2p^6^3d^n*^ electronic configuration can either be identical to the 2p^6^3d^*n*^ ground state, as in the case of elastic scattering, or correspond to an excited LF or 2p^6^3d^*n*+1^L* charge-transfer state arising from inelastic scattering. Resulting spectra are commonly presented on an energy transfer (valence excitation energy) scale relative to the elastic line.

The large spin–orbit coupling (SOC) of the 2p core-hole generated during the 2p3d RIXS process allows a change in one unit of spin-angular momentum for each excitation and relaxation process. As a result, the final observed states may be up to Δ*S* = 2 relative to the ground state.^23^ Additionally, the element selective nature of 2p3d RIXS means that only states involving the absorber of interest are observed, forgoing the issue of ligand-centered excited states that often otherwise obscure higher lying excited LF, ligand-to-metal charge transfer (LMCT), or MLCT states when probed using UV–vis/nIR spectroscopy. Furthermore, unlike the *C*-terms of MCD,^18^ 2p3d RIXS does not rely on paramagnetism and therefore is equally applicable for investigating the LF states of both diamagnetic and paramagnetic centers.^24,25^ Not least, unlike optical spectroscopies, LF states may be observed over a wide range of energies, where the low-energy limit is in principle restricted only by the width of the elastic scattering line, allowing transitions <1 eV to be observed. In recent years, these properties of 2p3d RIXS with moderate resolution (*E*/Δ*E* = 1000–5000) have been exploited to characterize the LF manifolds in a variety of coordination complexes.^23,26−29^

While 2p3d RIXS has the potential of providing *significantly* more LF information content than valence spectroscopic methods, metal ions with multiplet-rich manifolds (such as d^4^, d^5^, and d^6^ electronic configurations) and molecules with overlapping CT states can, conversely, produce sufficiently rich spectra that accurate assignment of spectral features can become highly challenging. In these cases, we can exploit the complementing selectivity of valence spectroscopic methods to deconvolute and analyze 2p3d RIXS. Furthermore, ab initio computational methods capable of providing first-principles quantitative descriptions of experimental spectra have the potential to provide perhaps the most extensive insights.

**Figure 1:**
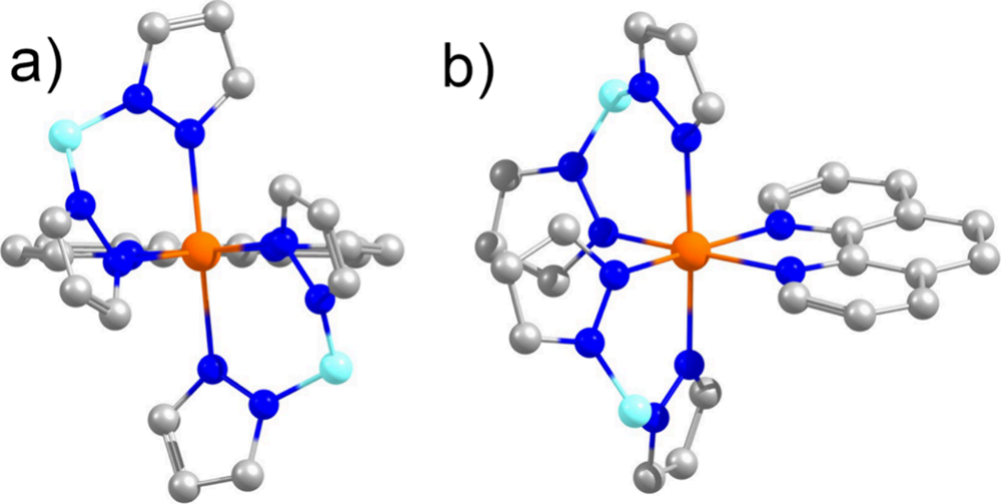
HS crystal structure [Fe^II^(H_2_B(pz)_2_)_2_phen]^0^ (a) along the *C*_*2*_ axis and (b) off-axis.

Herein, we have performed a systematic study of both HS and LS states of [Fe^II^(H_2_B(pz)_2_)_2_phen]^0^ (**1**, phen = 1,10-phenanthroline, H_2_B(pz)_2_ = bispyrazolylborate, Figure 1) with the goal of demonstrating the power using a multifaceted core/valence/computational spectroscopic approach to map the LF manifold of an SCO complex. We will (i) demonstrate the novel application of Fe 2p3d RIXS to a SCO system, (ii) demonstrate how the tandem employment of complementing valence and core-excitation techniques enhances insight into the electronic structure, particularly in discriminating charge transfer vs d-d (Δ*S* = 0) vs d-d (Δ*S* = 1) type transitions, (iii) demonstrate the exploitation of light-induced excited spin-state trapping (LIESST)/soft X-ray-induced excited spin-state trapping (SOXIESST) processes to investigate the ground and valence electronic structure of SCO complexes, (iv) present the novel application of CASCI/NEVPT2 ab initio calculations to characterize 2p3d excited states, and not least (v) examine how the LF properties of Fe are modulated through the SCO process.

## Materials and Methods

### Preparation of Materials

[Fe^II^(H_2_B(pz)_2_)_2_phen]^0^ (**1**) was synthesized and purified as previously described.^30^ The properties of SCO complexes are often quite sensitive to their state (solution vs solid) and physical handling, such as grinding or ball-milling. The ultrahigh vacuum environment required for Fe 2p3d RIXS makes the measurement of solutions, either liquid or frozen, particularly challenging. To maintain maximal continuity with the Fe 2p3d RIXS experiments, samples for UV–vis/nIR and MCD spectroscopic measurements were prepared as solids embedded in a polysiloxane mull. To make such mull samples optically viable, very mild grinding was required to thoroughly disperse the complex. To ensure sample integrity was maintained and the complex remained as a single species, low-temperature ^57^Fe Mössbauer spectroscopy was measured for the mull (see SI). These measurements demonstrate that, within experimental error (<1%), the mulling process does not produce additional spectroscopic species, such as a trapped HS state (Figure S1).

### Magnetization Measurements

Superconducting quantum interference device (SQUID) bulk magnetization measurements were performed for **1** using a Quantum Design MPMS-7 SQUID magnetometer calibrated with a standard palladium reference sample (error <2%). This sample consisted of randomly oriented microcrystals, with a total mass of 0.4 mg for **1**, which were placed in anaerobic quartz-glass holders for measurement. Additionally, as mechanical action such as grinding is well-known to influence the SCO behavior, a finely ground sample embedded in KBr was additionally measured using a sample containing 5% (by mass) **1** in KBr. Minute amounts of eicosane (melting point of 310 K) were used to prevent torquing of the material under large applied magnetic fields. The temperature dependence of the magnetic susceptibility was measured from 2 to 290 K at a magnetic field of 0.1 T. Blank measurements of the quartz-glass holder were performed for background subtraction. Desired magnetization data were acquired by fitting amplitudes from the appropriately corrected SQUID response curves. Values for the sample magnetization were divided by the number of moles and converted to units of molar susceptibility using the definition χ = μ_B_*M*/*B*. Values of χ were then corrected for any temperature-independent paramagnetism or underlying diamagnetism by the use of tabulated Pascal’s constants. The temperature-dependent magnetic susceptibility and field-dependent magnetization data were handled and simulated using the local software packages julX and julX_2S (developed by Dr. Eckhard Bill at the MPI-CEC, Mülheim an der Ruhr, Germany), which employ the spin-Hamiltonian described by eq 1 to calculate true powder averages in three dimensions, assuming the presence of an isolated spin ground state and collinear **D** and **g** matrices, where the **g** matrix may be isotropic, axial, or rhombic.
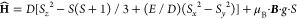
1

As *D* may be either positive or negative, values on both sides were explored. The value of *E* was constrained such that |*E*| < |*D*/3|. An averaged, isotropic *g*-tensor (*g*_av._) was employed in all modeling, in which
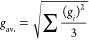
2Values for *E*, *D*, and *g* were optimized by a least-squares minimization using data from all fields and temperatures simultaneously.

### Optical Measurements

All presented optical measurements were performed on a finely dispersed powder in a silicon oil mull. The persistence of SCO behavior in this preparation medium was checked using a ^57^Fe Mössbauer, provided in the SI. UV–vis/nIR absorption measurements were performed using a Cary 6000i UV–vis/near-IR spectrophotometer in conjunction with an Oxford Instruments Optistat-DN cryostat at temperatures ranging from 80 to 220 K. Samples were prepared in a strictly anaerobic, moisture-free glovebox. Electronic absorption and variable-temperature variable-field (VTVH) MCD measurements were performed using an OLIS DSM17 UV–vis/nIR CD spectropolarimeter in conjunction with an Oxford instruments Spectromag SM4000 magnetocryostat. Spectra were recorded over the range of 300–800 nm using a photomultiplier tube detector and over the range of 700–2000 nm using an InGaAs detector. All low-temperature measurements were performed on samples prepared in MCD cells using 0.1 mM quartz windows. MCD and electronic absorption data were globally analyzed using local software (developed by E.B. at the MPI-CEC, Mülheim an der Ruhr, Germany). UV–vis/nIR (220 K) and LIESST-induced MCD spectra (3, 5, and 7 T at 5 K) were simultaneously fit in the energetic positions of a minimum number of Gaussian functions. The full-width half-maximum (fwhm) and intensity were allowed to vary, with the fwhm restricted to <2-fold difference between the narrowest and broadest features. Subsequently, UV–vis/nIR spectra collected at 80, 162, and 220 K were simultaneously fit using the same set of Gaussian functions (allowing intensity to vary), along with three additional Gaussian functions to account for the appearance of temperature-dependent spectral features at 80 and 162 K.

### LIESST

MCD and SQUID spectroscopies are dependent on the Boltzmann distribution of the magnetic ground state as determined by the Boltzmann equation, . Complex **1** has been demonstrated to exhibit LIESST behavior, whereby irradiation with light in the range of 647–676 nm of sufficient intensity induces LS → HS transition at very low temperatures, opening the possibility of significantly populating the electronic HS state while maintaining a favor Boltzmann distribution for magnetism-dependent methods.^30^ Characterization of the temperature and time-dependent behavior of the LIESST process in **1** has revealed that the photoinduced HS state appears relatively stable between 10 and 35 K but rapidly decays at a critical temperature of *T*_c,LIESST_ = 43 K.^30^ Therefore, in order to characterize the HS state of **1**, LIESST was utilized to saturate the HS state while remaining at temperatures <40 K. This was accomplished by illumination of samples using an LOT-XBO xenon lamp (300 W) from Ushio together with a fiber optic and a 640 nm low-pass filter. Assurance of saturation was accomplished by monitoring the signal intensity as a function of illumination time (see ESI, Figure S4). For SQUID, temperature and field-dependent measurements were performed under continuous illumination. As this condition was not possible for MCD, the sample was reilluminated for 15 min between each individual scan to ensure HS state saturation. During this time course, an estimated 3% decay was calculated by remeasurement of MCD intensity at 600 nm.

### X-ray Measurements

All soft X-ray measurements were performed at the AERHA end station of the SEXTANTS beamline at the SOLEIL synchrotron facility.^31^ This beamline provides monochromatic incident X-rays with a resolving power (Δ*E*/*E*) greater than 1 × 10^4^. Linear horizontal polarization was employed to suppress elastic scattering. The angle between the incident and scattered beams was fixed at 85°, with the sample fixed at 45.7° relative to the incident beam. The incident beam spot size was approximately 2 × 100 μm (*v* × *h*) in normal incidence, and the final energy resolution was estimated to be ∼310 meV, as determined by the FWHM of the Voigtian fit of the elastic line. The X-rays emitted from the sample were dispersed in the vertical direction by a variable line density plane grating and collected on a position-sensitive detector (CCD).

The Fe L-edge spectra of the HS and LS states of **1** were used to select the incident excitation energies for the 2p3d RIXS measurements. XAS spectra were collected as total electron yield measurements via the drain current from the sample holder. The energy of the beamline monochromator was calibrated using the spectrum of Fe_2_O_3_, where the maximum of the L_3_-edge was set to 708.5 eV. As the molecular samples are quickly damaged at soft X-ray RIXS beamlines due to the combination of high incident flux over small spot sizes, samples were continuously rastered during measurement to avoid beam damage. Vertical scan rates of 20, 50, 100, 200, and 400 μm/s were tested (corresponding to sample exposure times of 0.1, 0.04, 0.02, 0.01, and 0.005 s, respectively, given the 2 μm beam height), both with and without the presence of additional Co filters, which further reduced the incident flux by ∼100-fold. Final movement speeds were chosen that resulted in no spectral changes relative to faster scan speeds.

The high-temperature (200 K) spectrum of **1** was found to be relatively insensitive to X-ray radiation, consistent with the fact that Fe^II^ samples are generally resistant to photoreduction. These spectra were concluded to be representative of the HS state, and scan rates of 20 μm/s were used for data collection at 200 K. Surprisingly, the low-temperature (∼20 K) spectra were found to be much more sensitive to X-ray exposure. In fact, it was found that the low-temperature XAS spectrum rapidly converts to the 200 K spectrum with the HS spectrum observed in the RIXS even with 400 μm/s scan rates (Figure S7). This behavior, referred to as SOXIESST, is reversible and has been previously observed in Fe L-edge XAS measurements for several SCO systems.^32−35^ Upon inserting a Co filter (available at SEXTANTS) to reduce the incident flux on the sample and increasing the sample temperature (65 K) to above the trapping temperature (*T*_c,LIESST_), we could measure a distinct XAS spectrum. This is assigned to the LS state, and LS RIXS spectra were acquired under these conditions using scan rates of 20 μm/s.

### CASSCF/NEVPT2 and CASCI/NEVPT2 Calculations

All calculations were performed using the quantum computing suite ORCA 4.2.1.^36^ As all spectroscopic experiments were performed using the solid complex, all calculations employed the reported crystal structures for HS and LS forms collected at room temperature and 30 K,^37,38^ respectively, with optimization steps only being performed for hydrogen atoms. Geometry optimizations of hydrogen atoms were performed at the level of density functional theory with the pure exchange correlation functional BP86, which utilizes Becke’s 1988 exchange functional^39^ along with the gradient corrections of Perdew as well as Perdew’s 1981 local correlation functional,^40^ in conjunction with the second definition triple-ζ valence polarized (def2-TZVP) basis of Ahlrichs and co-workers and the complimenting def2/J auxiliary basis set.^41−43^

Calculations of ground- and excited-state properties were performed using the state-averaged complete-active-space self-consistent field (SA-CASSCF) method^44,45^ with consideration of SOC using quasi-degenerate perturbation theory in conjunction with N-electron valence perturbation theory to second order (NEVPT2)^46^ in order to recover missing dynamic electron correlation, similar to previous studies.^47^ These calculations were performed using the relativistic triple-ζ atomic natural orbital basis dkh-def2-TZVP along with the AutoAux basis option of ORCA,^48^ in conjunction with second-order Douglas-Kroll-Hess (DKH2)^49^ Hamiltonian to account for scalar relativistic effects. SOC was treated through the mean field (SOMF) approximation,^50,51^ and the effective Hamiltonian approach^52−54^ was used to compute SH parameters.

To more accurately model the LF structure and optical spectroscopic data, several active spaces were chosen. Active spaces are denoted as (X,Y), where X is the number of electrons and Y is the number of orbitals. First, a minimal (6,5) active space with six electrons in the five valence Fe 3*d*-centered orbitals was chosen. Second, this minimal space was expanded to (6,10) to include the 4*d*-shell, which is known to improve estimates of electronic correlation. Third, the complementing bonding σ-interactions between Fe and the surrounding ligand were considered in a (10,12) active space. Last, both complementing bonding σ- and π-interactions of the surrounding ligand system were added to the Fe-3d-antibonding orbitals to generate a (16,10) active space. In the HS case, the expanded geometry (Fe–N_avg_ ∼ 2.2 Å) results in a lower degree of ligand/metal overlap for the Fe 3*d*_*xy*_, making this orbital effectively nonbonding. Therefore, the largest HS active space was limited to (14,9). A total of 50 singlet, 45 triplet, and 5 quintet roots were calculated for all active spaces in both HS and LS complexes to account for all LF excited states. The AILFT analysis was requested for the (6,5) active space by canonicalizing the active orbitals in a specific manner (keyword actorbs = dorbs in ORCA).^55,56^

To model Fe L_2,3_-edge XAS spectra and subsequent resonant inelastic X-ray scattering spectra following the CASCI/NEVPT2 protocol for calculating core excited states,^57,58^ active spaces (12,8) and (22,13) were utilized for both HS and LS complexes by using a two-step procedure. First, the (6,5) and (16,10) active spaces described above were optimized using 50 singlet, 45 triplet, and 5 quintet roots. In the second step, the 2p orbitals were rotated into the active space to generate (12,8) and (22,13) active spaces. Subsequently, a saturating number of single, triple, and quintet roots was calculated involving all core-level single excitations and valence level single and double excitations. Additionally, for the (22,13) active spaces, single excitations from the bonding orbitals were also considered. For (12,8), this involved 135 singles, 125 triples, and 35 quintets. For (22,13), this involved 735 singles, 700 triples, and 335 quintets. Despite previous studies showing little impact of Δ*S* = 2 transitions on Fe L_2,3_-edge XAS spectra,^59^ these states were calculated for consistency in the LF descriptions of either HS or LS complexes.

### LF Analysis

The program AOMX^60^ was utilized for the analysis of all data in terms of LF parameters. AOMX calculates the *d*^*n*^ electron terms in the framework of either the crystal-field model or the angular overlap model. The ability to fully determine the LF parameters of a given complex experimentally is highly dependent upon the number of available observable electronic transitions and choice of their assignment. In practice, one is typically limited to a low-energy subset of spin-allowed LF transitions, such that the problem usually requires more parameters than can be determined by the available information. Meanwhile, calculations can provide all LF states of interest. The discrepancy in available information has often led to considerable disagreement between calculated and experimentally determined parameters. In particular, a lower number of experimental points requires a greater number of assumptions to be made in the applied interpretive model. Therefore, LF parameters determined from CASSCF/NEVPT2 and CASCI/NEVPT2 calculated excited states were derived using the same excited states as assigned experimentally. For more details on the calculation of LF parameters, please see the SI Section 1.

## Results

To compare the observable LF excited state of **1** in both HS and LS from both valence- and core-excitation perspectives, a series of spectroscopic methods were employed. To examine valence excitations, UV–vis/nIR and MCD spectroscopies were used, while the core Fe L_3_-edge excitation was used to further probe excited states in the same energy range through Fe 2p3d RIXS, which takes advantage of a resonant dipole-allowed excitation/emission process to access LF [formally (Δ*S* = 0, 1, 2)] and other predominately metal-centered excited states. Furthermore, the LIESST behavior of complex **1** allowed the ^5^T “ground” state magnetic properties of **1** to be investigated. Not least, CASCI/NEVPT2 calculations of **1** were performed to complement our experimental findings.

### SQUID

Variable-temperature SQUID measurements demonstrating the SCO behavior of **1** have been previously reported, which revealed sharp spin transitions centered at 161.8 and 165.6 K (on cooling and warming, respectively), with 70% of the spin conversion occurring within an interval of 6 K.^37^ These properties have been reproduced currently (Figure S2). Additionally, previous ^57^Fe Mössbauer studies have demonstrated 100% LS at below 80 K, and >85% HS above 250 K.^37^ By taking advantage of the previously established LIESST^61,62^ properties of **1**,^37^ we irradiated **1** using 637–676 nm wavelength light to saturate the HS state which has been shown to be relatively stable below the critical relaxation temperature *T*_c,LIESST_ = 43 K.^30^ In this way, variable-temperature measurements were performed at 1 T across a range of low temperatures (from 2 to 35 K) to characterize the magnetic properties of the HS state of **1** (Figure S3). Table 1 summarizes the fit effective spin-Hamiltonian parameters of the LIESST-induced HS state of **1**. The *S* = 2 magnetic ground state can be reasonably simulated using an effective spin-Hamiltonian with relatively large, positive zero-field splitting (ZFS) of *D* = 13.7 cm^–1^, high rhombicity (*E/D* = 0.33), and *g*_avg_ = 2.33.

**Table 1:** Summary of the Spin-Hamiltonian Parameters of the LIESST-Induced HS State of **1**, as well as the SA-CASSCF/NEVPT2 Calculated Values

	*g*_avg_	*D* (cm^–1^)	*E/D*
mag. susceptibility	2.33	13.7	0.33
VTVH-MCD	2.12	10.7	0.21
(6,5)CASSCF/NEVPT2	2.18	21.0	0.12
(6,10)CASSCF/NEVPT2	2.18	19.9	0.13
(16,10)CASSCF/NEVPT2	2.18	23.7	0.24

### UV–Vis and MCD

**Figure 2:**
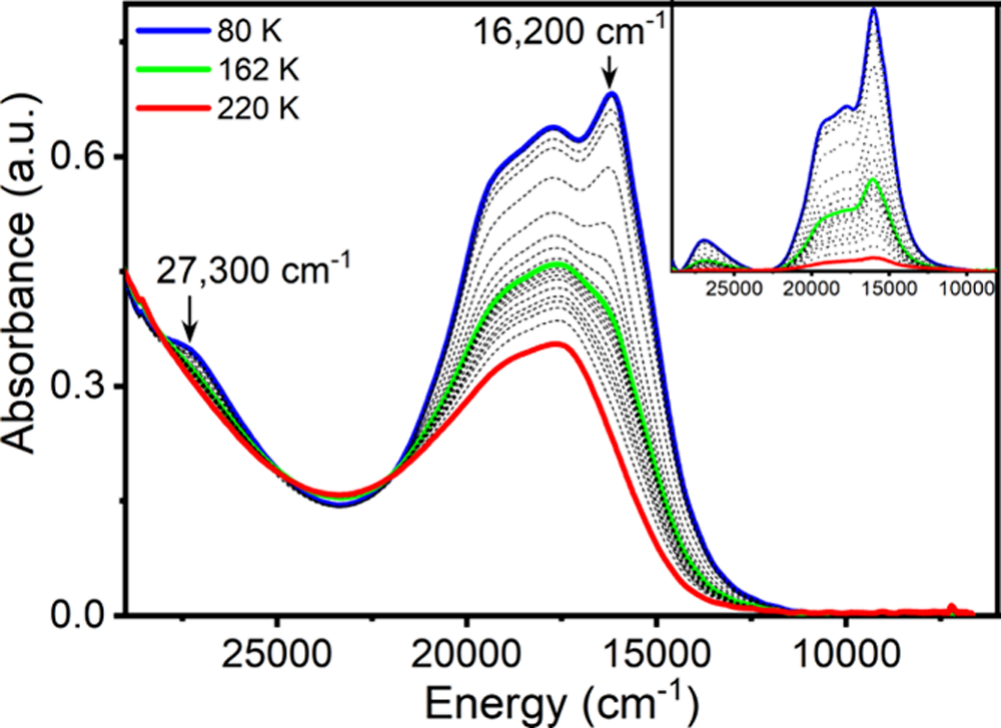
Temperature-dependent UV–vis spectrum of **1** imbedded in a polysiloxane mull. The inset shows the temperature-dependent difference spectra, with the 220 K spectrum, shown in (a), subtracted.

The temperature-dependent UV–vis/nIR spectrum of **1** was collected to explore the spectral changes that occur by moving between the HS and LS states. The resulting spectra are listed in Figure 2. The low-energy region (12,000–20,000 cm^–1^) displays significant temperature dependence, increasing in total absorption nearly 2-fold as a function of decreasing temperature across the range examined. The HS state (220 K) exhibits a broad feature with two inflections at ∼17,600 and ∼19,500 cm^–1^. The estimated extinction coefficient of this feature appears in the range of ε = 1000–2000 cm^–1^, indicative of charge-transfer type transitions. A precise determination of the extinction coefficient was not possible due to the uncertainties associated with the precise determination of both concentration and thickness for solid-state samples. As the temperature is lowered, the complex shifts in color from purple to deep blue, and these two features increase in intensity. Additionally, two new features appear at ∼16,200 and ∼27,300 cm^–1^, reaching maxima at the lowest measured temperature of 80 K. Based on magnetic susceptibility measurements (Figure S2), the complex is anticipated to be nearly completely LS at this temperature.

Although two new features appear in the UV–vis moving from HS to LS that appear as viable candidates for the spin-allowed LF states of LS Fe^II^, no noticeable spectral changes are observed in the anticipated energetic region of the only spin-allowed HS Fe^II^ LF transition, ^5^T → ^5^E (9000–13,000 cm^–1^).^63^ However, LF transitions are often extremely weak and not always detectable using standard optical absorption spectroscopy. Therefore, we further employed MCD spectroscopy to investigate the optical and magnetic properties of HS **1**.

**Figure 3:**
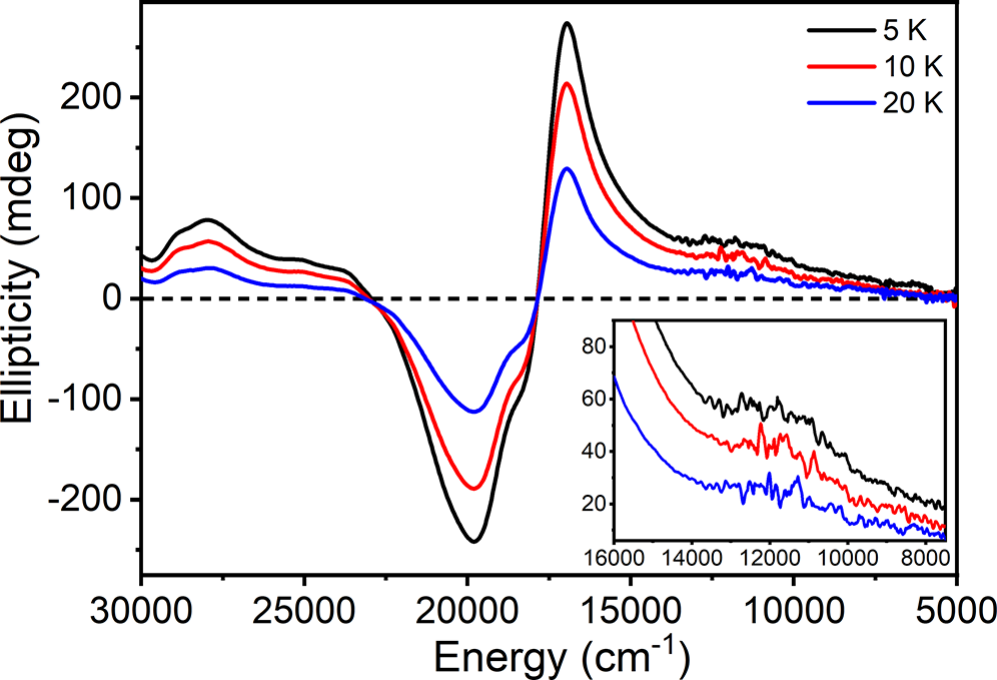
Temperature-dependent MCD of HS **1** embedded in a polysiloxane mull. Spectra were collected at an applied field of 5 T. The HS state was generated by exposure of the sample to light irradiation (640 nm) until the signal intensity at 590 nm was saturated. Re-exposure to light irradiation was performed between individual scans of the field and temperature. A moving average smoothing with an 11 point width was performed over the region of 5000–14,000 cm^–1^. An equivalent plot without filtering is provided in the ESI, Figure S5.

Briefly, MCD measures the difference between left and right circularly polarized light as induced by the presence of a strong magnetic field oriented parallel to the direction of light propagation that features gain intensity through the Zeeman effect, which includes the magnetic perturbation of electronic states involved in light absorption. Absorption features are classified in three terms (*A, B, C*) depending on their nature of origin. *A*-terms involve electronic transitions from electronically degenerate states to/from electronically degenerate states. *B*-terms arise from electronic mixing between nondegenerate states and are typically weak compared to *A-* or *C*-terms. Systems with ground-state degeneracy (either electronic or magnetic) will produce 1/*T* temperature-dependent *C*-terms, where the spectral intensity will rely on the Boltzmann distribution of the ground state. As high symmetry systems are relatively rare, MCD is commonly employed to selectively investigate paramagnetic systems that display *C*-terms originating from the magnetic degeneracy of the ground state. However, the application of MCD to studying Fe^II^ SCO systems presents a unique challenge as the *C*-term intensity of paramagnetic ions is inversely proportional to temperature, and therefore very weak at temperatures that thermally access the HS state.^25^ This is similar to the obstacle faced in applying magnetic susceptibility measurements to probe the HS state. To overcome this barrier, we again employ LIESST to trap the HS at temperatures below *T*_c,LIESST_ (Figure S4). In this way, MCD was collected for the LIESST-saturated *S* = 2 HS state across a range of 5, 10, and 20 K at 5 T (Figure 3).

The HS state of **1** exhibits a rich and intense MCD spectrum dominated by a large negative *pseudo A-*term centered at ∼17,500 cm^–1^. All observed features appear temperature dependent and are therefore assigned as *C*-terms. As the MCD *C*-term intensity is dependent on spin-angular momentum, only transitions with significant Fe^II^ character in either the ground or excited state (or both) are anticipated to significantly contribute to the spectrum.^17−20^ Therefore, the considerable intensity of features in the 15,000–22,000 cm^–1^ range are indicative of significant ligand/metal charge-transfer character, consistent with UV–vis measurements. While the low-energy region from 5000 to 15,000 cm^–1^ exhibits a broad, sloping background, a weak positive temperature-dependent feature is apparent at ∼11,700 cm^–1^. We note that, to the best of the author's knowledge, this is the first demonstration of the use of MCD to investigate the valence electronic structure of a SCO complex.

VTVH-MCD measurements were performed across temperatures of 5, 10, and 20 K at 16,950 cm^–1^ to provide a cross-correlation with magnetic susceptibility measurements (Figure S6). Results of the effective spin-Hamiltonian fits are listed in Table 1. Fits were performed starting from values determined by magnetic susceptibility measurements and refined to best fit the data. A reduction in *g*_avg_ to 2.12 is required, as well as a slightly smaller *D* = 10.7 cm^–1^ and a reduced degree of rhombicity (*E/D* = 0.21). This transition was also best fit as *x,y* polarized.

### Fe L_3_-Edge XAS and 2p3d RIXS

**Figure 4:**
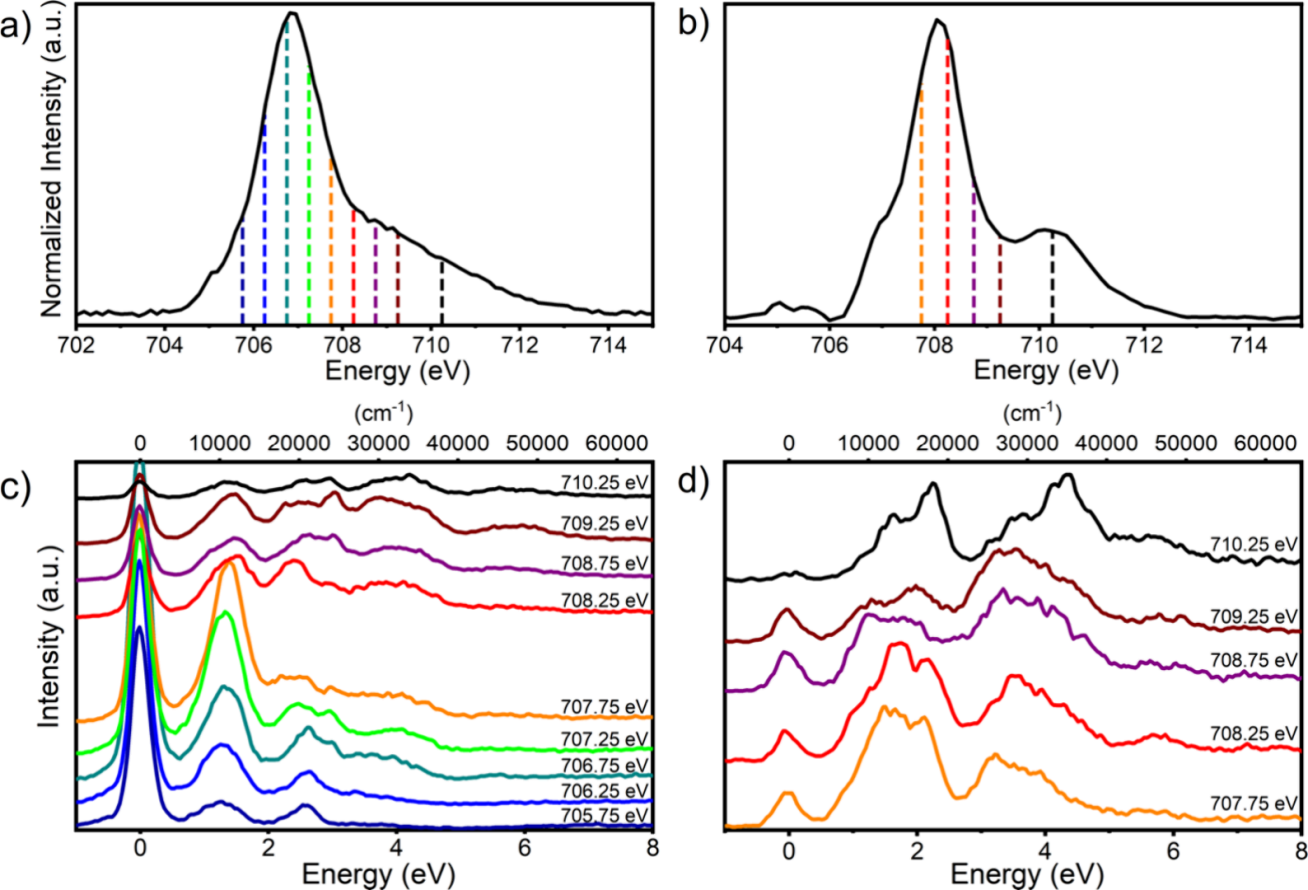
Fe L_3_-edge absorption spectra of the (a) HS and (b) LS states of **1**^0^ acquired at 220 and 50 K, respectively. (c) Fe 2p3d RIXS acquired at 220 K and (d) 50 K, collected at the indicated incident energies. RIXS spectra are color-coded with drop lines placed on the corresponding L_3_-edge spectra to indicate incident energies.

The Fe L_3_-edge XAS spectra of HS and LS **1** are provided in Figure 4a,b. Both spectra are relatively featureless, dominated by a single major feature centered at 706.8 eV (HS) and 708.0 eV (LS). The dominant feature of the L_3_-edge is particularly broad in the HS case, with an apparent fwhm of 2 eV. While electronic states involving both 2p → 3d(t_2g_*) and 2p → 3d(e_g_*) transitions are possible in HS Fe^II^, states involving these transitions can be difficult to resolve in systems with relatively small crystal field, (10*D*_q_ < 2 eV). Additionally, the HS spectrum exhibits a small satellite feature at 705.0 eV broad, and a broad sloping tail from 708.5 to 712 eV.

The LS state is anticipated to have a significantly greater crystal-field splitting than the HS state (presumably 10Dq > 2 eV); therefore, the fully occupied t_2g_ orbitals of octahedral Fe^II^ preclude electronic states involving 2p → 3d(t_2g_*) transitions. As a result, only a single major feature is expected, arising from states involving the 2p → 3d(e_g_*) transition. The energy of this feature is also expected to be shifted to higher energy relative to the HS case due to 1) the symmetric contraction of metal–ligand bond distances which results in an increased σ–σ* interaction between the 3d(e_g_*) and surrounding ligands, and 2) the absence of the lower energy 2p → 3d(t_2g_*) component. Indeed, this is observed in the LS spectrum (Figure 4b), where the dominant feature at 708.0 eV exhibits a smaller fwhm (∼1.2 eV) and is shifted to a higher energy by +1.2 eV relative to the HS case. This energy shift is similar to that observed between the HS and LS states of other Fe^II^ complexes, such as [Fe^II^(phen)(NCS)_2_]^0^ (1.4 eV) and [Fe^II^(tren)(py)_3_]^3+^ (1.7 eV).^33,34,64−66^ The LS spectrum also displays several additional features. At the lower energy side, a weak pre-edge feature around 705.2 eV is observed, along with a shoulder around 707 eV. At higher energy, a peak at 710.2 eV appears; while previous studies of Fe^II^ complexes that have exhibited this high-energy feature have assigned this as a MLCT feature,^67^ these intense features can also arise from higher LF states with strong double excitation character, 2p → 3d(e_g_*) + 3d(t_2g_*) → 3d(e_g_*).

RIXS measurements were performed to further analyze the LF properties of **1** in both HS and LS states. Spectra are provided in Figure 4 and labeled with corresponding incident energies, as indicated on plots of the corresponding L_3_-edge spectra. To avoid sample damage by the soft X-rays used in RIXS, low temperatures are generally required, typically 20 K. However, irradiation with soft X-rays can induce SOXIESST, as described in the methods section, which is proposed to proceed via the injection of X-ray induced secondary electrons.^35^ Presently, Fe L_3_-edge XAS and 2p3d RIXS measurements of **1** performed at temperatures below *T*_c*,*LIESST_ exhibited SOXIESST behavior (Figure S7). By raising the sample temperature above *T*_c*,*LIESST_ while remaining well below the thermal SCO temperature *T*_c_, we could observe L-edge spectra consistent with LS Fe^II^, similar to observations for [Fe(phen)_3_](NCS)_2_.^34^

Transition-metal L-edge RIXS spectra are generally dominated by local LF transitions, which will vary significantly in HS vs LS systems in terms of both energy and intensity due to large changes in the LF strength (10D_q_) that accompanies the HS/LS transition. For both spin states, the RIXS spectra appear as roughly three to four groups as a function of energy transfer. In the HS, these groups appear between 0.7 and 1.9 eV (5600–15,300 cm^–1^), 1.9–3.2 eV (15,300–25,800 cm^–1^), 3.2–5 eV (25,800–40,300 cm^–1^), and >5 eV (Figure 4c). In the LS case, these groups appear broader, ranging from 0.5 to 2.7 eV (4000–21,800 cm^–1^), 2.7–5 eV (21,800–40,300 cm^–1^), and 5–6.8 eV (40,300–54,800 cm^–1^) (Figure 4d).

Beyond the significant changes in RIXS spectral shapes observed for HS vs LS, inspection of either HS or LS spectra in Figure 4 clearly displays that choice of incident energy results in heavy modulation of the RIXS in terms of both energy and intensity. While the energetic *position* of LF states relative to the elastic line is independent of incident energy, the relative population of final states can vary significantly, leading to large spectral differences in terms of the number of observed inflections, their energetic positions, and their intensity. Therefore, interpreting and deconvoluting the spectra obtained at multiple incident energies *simultaneously as a group*, rather than individually, significantly enhances the resolution and therefore the information content. This is discussed further below.

### Theoretical Calculations

**Figure 5:**
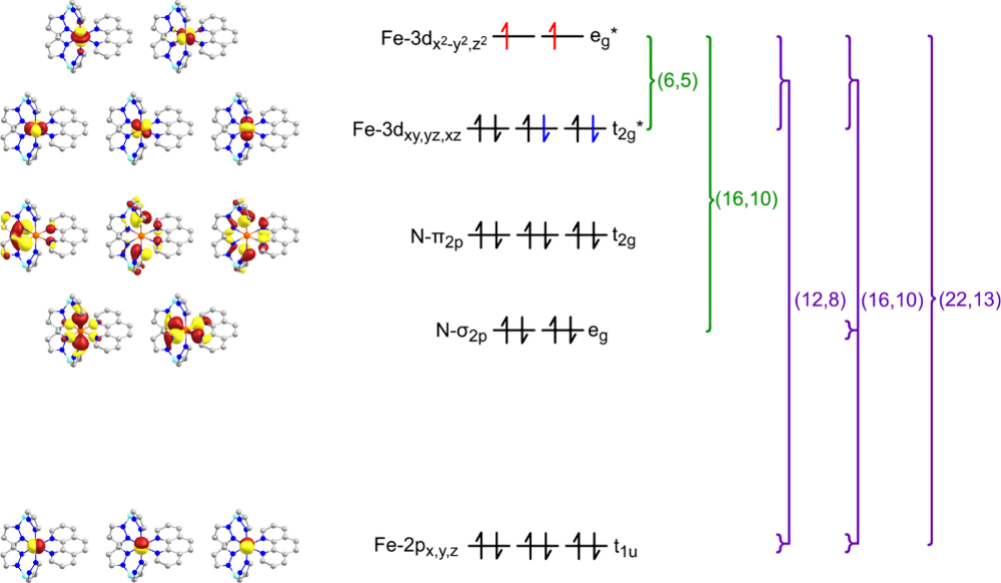
Molecular orbital diagram, including isosurfaces (left), orbital occupation patterns (center) with atom-localized labeling (left), and symmetric labeling (right), and examples of MO grouping used in CASSCF/NEVPT2 (green) and CASCI/NEVPT2 (purple) calculations. Molecular orbital surfaces (left) are plotted using a 0.05 isovalue for all valence orbitals (Fe-3d and N) and 0.0001 for core orbitals (Fe-2p). Orbital occupation patterns are provided in black/red for HS and black/blue for LS.

Ab initio calculations were performed at the SA-CASSCF/NEVPT2 and SA-CASCI/NEVPT2 levels with varying active spaces to calculate the valence, L_2,3_-edge, and 2p3d RIXS properties of both HS and LS states. The compositions of varying active spaces explored are listed in Figure 5. For valence excited-state calculations performed by CASSCF/NEVPT2, the minimal (6,5) active space containing only 3d-centered orbitals/electrons was additionally expanded to (16,10) to include σ and π-bonding interactions. These active spaces were further expanded from (6,5) → (12,8) and (16,10) → (22,13) by inclusion of the Fe-2p orbitals to allow for the calculation of the L_2,3_-edge and 2p3d RIXS by CASCI/NEVPT2. Additionally, a (16,10) active space including the 3d-centered orbitals, respective σ-bonding interactions, and core Fe-2p orbitals was investigated. The discussion of the electronic structure will be relegated to an O_h_ approximation to match our experimental resolution.

### Ground-State Properties

While the LS state is *S* = 0 (and therefore no ZFS), the calculated HS state is found to exhibit *g*_avg_ = 2.18 and ZFS parameters *D* = 21–23.7 cm^–1^ and *E/D* = 0.12–0.24 (also provided in Table 1). Calculated values of *g*_avg_ are within the range derived from the experiment, and the axial ZFS parameter *D* is overestimated for all calculations, with dominant contributions coming from the second (∼13 cm^–1^) and third (∼6 cm^–1^) roots of the quintet ground state. Interestingly, inclusion of σ and π-bonding interactions by expanding to the more complete (16,10) active space significantly improves the rhombicity parameter *E*/*D*, reflective of the relatively low (C_2_) true symmetry of the system as described in the SI. Analysis of individual state contributions to the computed ZFS values indicate that the main contributions arise from first two excited subcomponents of the parent ^5^T ground state (both ^5^B under *C*_2_ symmetry), which are split by ∼350 and ∼750 cm^–1^, varying slightly depending on the active space composition. It should be noted that such “near state degeneracy” condition implies challenges and a dynamic nature in the computed ZFS.^68,69^ Hence, a comprehensive computation would require proper inclusion of spin-phono coupling effects, which is beyond the scope of the current work.

### Fe L_3_-Edge Calculations

**Figure 6:**
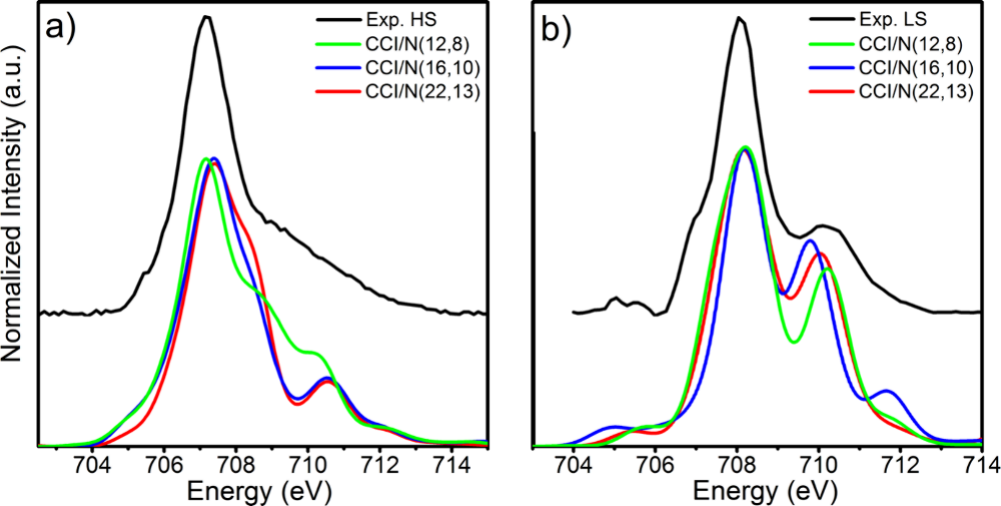
Comparison of experimental and SA-CASCI/NEVPT2 calculated Fe L_3_-edge absorption spectra of the (a) HS and (b) LS states of **1**. Experimental spectra were acquired at 220 K (HS) and 50 K (LS), respectively. Uniform energetic shifts of −0.7 and −0.55 eV have been applied to the HS and LS spectra, respectively.

The SA-CASCI/NEVPT2 calculated HS and LS Fe L_3_-edge spectra are provided in Figure 6. The experimental HS L_3_-edge is composed of a broad dominant feature centered at 706.8 eV with a fwhm of 1.8 eV. Additionally, a very broad tail spanning 708.3–713.5 eV is observed. The SA-CASCI/NEVPT2-computed HS spectra are all shifted by −0.7 eV to account for the error in the calculation of the absolute energy of the L_3_-edge. Computed spectra display up to four features: (1) a dominant peak ∼707.3 eV, a shoulder ∼708.6 eV, a second peak ∼710.7 eV, and finally a higher energy feature at 712.5 eV. Meanwhile, the LS L_3_-edge displays a main feature at 708.2 eV (FWHM = 2 eV), with a weak lower energy feature ∼705 eV and a single higher energy feature at 710.3 eV. Similar to the HS spectra, the calculated LS spectra overestimate the edge energy, and a −0.55 eV shift is applied.

### Valence Electronic States and RIXS calculations

**Figure 7:**
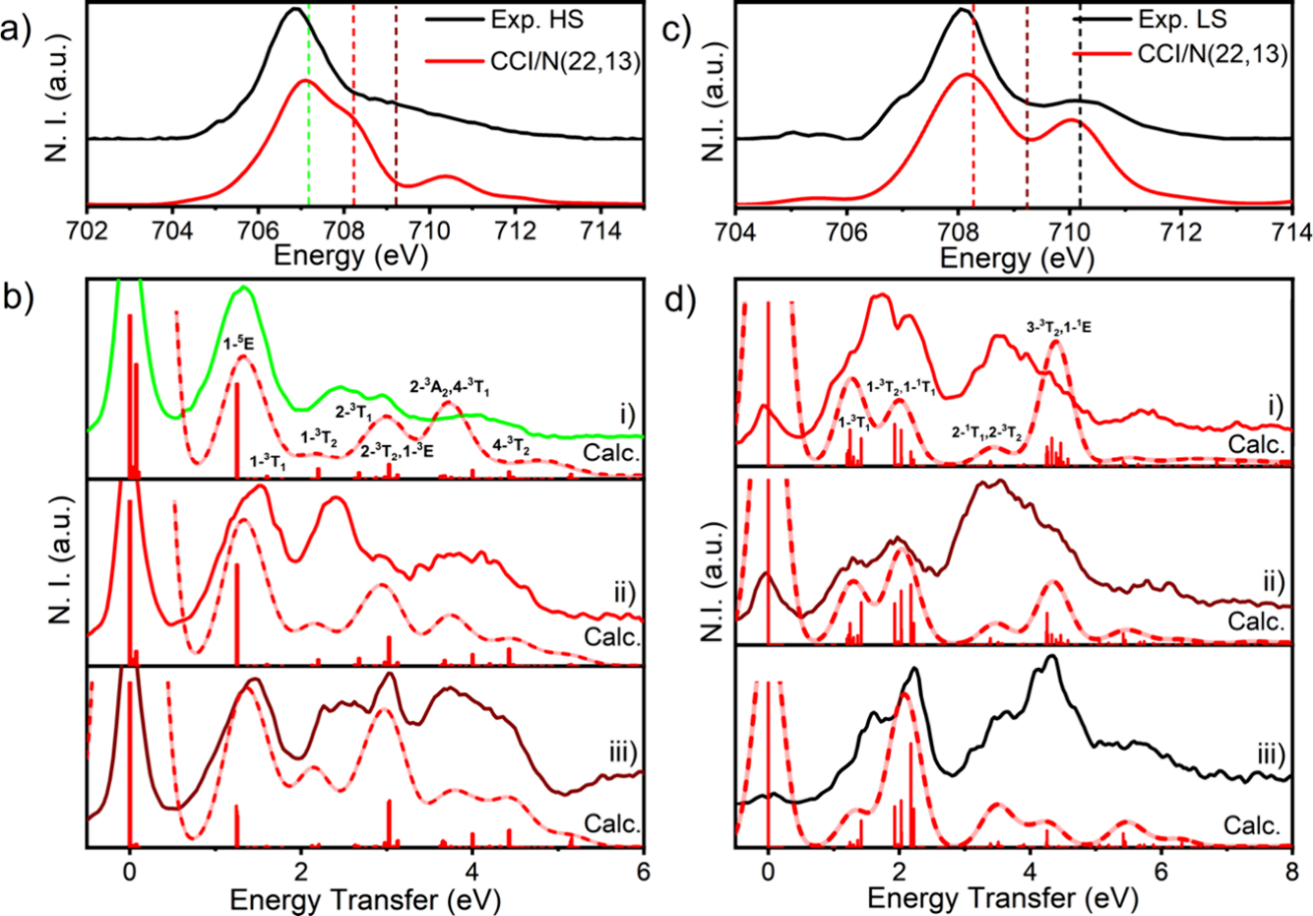
Comparison of the experimental and SA-CASCI/NEVPT2 calculated Fe 2p3d RIXS. (a) Experimental HS vs SA-CASCI/NEVPT2(22,13) calculated Fe L_3_-edge; (b) HS experimental and calculated RIXS from incident energies at (i) 707.25, (ii) 708.25, and (iii) 709.25 eV. (c) Experimental LS vs SA-CASCI/NEVPT2(22,13) calculated Fe L_3_-edge; (d) LS experimental and calculated RIXS from incident energies at (i) 708.25, (ii) 709.25, and (iii) 710.25 eV. Uniform energetic shifts of −0.7 eV and −0.55 eV have been applied to the calculated HS and LS spectra shown in (a,c), respectively, as well as to the selected incident energies used in selecting displayed calculated RIXS spectra.

The SA-CASCI/NEVPT2 calculated LF excited states of the HS and LS **1** from 0 to 40,000 cm^–1^ and 0 to 50,000 cm^–1^ are provided in Tables 2 and 3. To ease the comparison between X-ray and optical data as well as existing literature, transition energies are provided in both cm^–1^ and eV. Additionally, excited-state energies calculated at the SA-CASSCF/NEVPT2 level are provided in Tables S1 and S2. Although **1** is not rigorously O_h_ in symmetry, we have averaged the calculated excited states as such due to their sheer number and small splitting relative to experimental resolution. Comparisons of the calculated CASCI/NEVPT2 RIXS and corresponding experimental spectra are shown in Figure 7.

**Table 2:** Summary of SA-CASCI/NEVPT2 Calculated LF States of HS **1** below 40,000 cm^–1^, Averaged for an O_h_ LF

state	active space					
	(12,8)		(16,10)		(22,13)	
	eV	cm^–1^	eV	cm^–1^	eV	cm^–1^
1-^5^T_2_	0	0	0	0	0	0
1-^5^E	1.41	11,390	1.32	10,620	1.32	10,660
1-^3^T_1_	1.49	12,030	1.59	12,810	1.57	12,640
1-^1^A_1_	1.48	11,960	1.81	14,600	1.79	14,430
1-^3^T_2_	2.12	17,090	2.18	17,590	2.17	17,490
2-^3^T_1_	2.71	21,890	2.70	21,790	2.69	21,710
1-^1^T_1_	2.71	21,890	2.85	23,000	2.84	22,870
2-^3^T_2_	2.98	24,020	2.99	24,110	2.98	24,030
1-^3^E	3.08	24,880	3.07	24,740	3.06	24,650
3-^3^T_1_	3.14	25,330	3.12	25,180	3.11	25,120
3-^3^T_2_	3.72	30,020	3.67	29,620	3.66	29,560
1-^1^T_2_	3.71	29,890	3.76	30,360	3.75	30,260
1-^3^A_2_	3.84	30,990	3.77	30,400	3.76	30,340
2-^1^A_1_	4.08	32,890	3.86	31,150	3.85	31,070
4-^3^T_1_	4.09	33,020	4.00	32,270	4.00	32,270
2-^3^E	4.21	33,970	4.12	33,240	4.11	33,220
1-^3^A_1_	4.50	36,310	4.17	35,910	4.16	33,530
1-^1^E	3.82	30,830	4.27	33,650	4.17	33,600
2-^1^T_2_	4.27	34,400	4.45	34,420	4.26	34,360
4-^3^T_2_	4.58	36,930	4.51	36,380	4.47	36,090
2-^1^T_1_	4.67	37,680	4.63	37,320	4.62	37,260
1-^1^A_2_	4.78	38,570	4.72	38,130	4.72	38,090

**Table 3:** Summary of SA-CASCI/NEVPT2 Calculated LF States of LS **1** below 50,000 cm^–1^, Averaged for an O_h_ LF^a^

state	active space					
	(12,8)		(16,10)		(22,13)	
	eV	cm^–1^	eV	cm^–1^	eV	cm^–1^
1-^1^A_1_	0	0	0	0	0	0
1-^3^T_1_	1.05	8430	0.84	6,780	1.25	10,040
1-^5^T_2_	0.63	5080	0.23	1,830	1.30	10,470
1-^3^T_2_	1.83	14,740	1.58	12,770	1.98	15,960
1-^1^T_1_	2.20	17,770	2.01	16,220	2.19	17,680
2-^3^T_1_	3.22	25,940	2.82	22,750	3.39	27,330
2-^3^T_2_	3.42	27,610	3.04	24,520	3.52	28,430
1-^1^T_2_	3.63	29,250	3.32	26,820	3.54	28,530
1-^5^E	3.02	24,330	2.47	19,990	3.55	28,600
1-^3^E	3.65	29,510	3.25	26,210	3.82	30,790
3-^3^T_1_	3.68	29,700	3.27	26,410	3.88	31,300
3-^3^T_2_	4.28	34,540	3.87	31,250	4.26	34,430
1-^1^E	4.45	35,920	4.09	32,970	4.36	35,160
2-^1^T_2_	4.51	36,420	4.15	33,460	4.46	36,010
2-^1^A_1_	4.55	36,730	4.17	33,610	4.57	36,900
1-^3^A_2_	4.83	38,990	4.34	35,030	5.05	40,760
2-^1^T_1_	5.34	43,110	4.91	39,610	5.22	42,110
1-^1^A_2_	5.52	44,560	5.08	40,970	5.42	43,690
2-^1^T_2_	5.67	45,740	5.27	42,500	5.46	44,030
3-^3^T_1_	5.70	46,010	4.99	40,280	5.64	45,530
2-^3^E	5.87	47,380	5.11	41,200	5.68	45,820

a States assigned to observed spectral features are emphasized in bold.

Despite a multitude of final excited states in the calculated HS and LS RIXS, our CASCI/NEVPT2 calculations reveal that relatively few significantly contribute to the overall spectra. Specifically, although the large SOC of the 2p core-hole allows for Δ*S* = 1 transitions upon excitation and again Δ*S* = 1 upon relaxation, in principle, enabling an intensity mechanism for the appearance of Δ*S* = 2 states, these make little contribution to the calculated spectra of either the HS or LS state. This is consistent with our previous findings for LS Fe.^23^

The states listed in Tables 2 and 3 are prefixed numbers (1, 2, 3) to distinguish states with identical symmetry labels based on energetic ordering, with 1- denoting the lowest energy. The HS spectra are dominated by the 1-^5^E excited state at low energy (∼1.3 eV), with all other significant contributions arising from ^3^T states (Figures S8–S11). The 1-^3^T_2_ and 2-^3^T_1_ states consistently appear between 2.1 and 2.2 eV (17000–17600 cm^–1^) and around 2.7 eV (21,800 cm^–1^). The pronounced 2-^3^T_2_ state is calculated at ∼3.0 eV (24,100 cm^–1^), with the 2-^3^A_2_, 4-^3^T_1_, and 4-^3^T_2_ constituting most of the spectral intensity between 3 and 5 eV (24,000–40,000 cm^–1^). Between the three investigated active spaces, relatively little energetic variation (<3000 cm^–1^) is found for most calculated HS states. For the HS CASCI/NEVPT2(22,13) RIXS cuts, we observe a significant dependence on the choice of excitation energy. This is perhaps clearest when comparing the cuts presented in Figure 7b, where the relative intensity of features arising from the relative intensity of states 2-^3^T_2_,1-^3^E:2-A_2_,4-^3^T_1_:4-^3^T_2_ appear significantly improved at higher excitation energies. We note that a significant lack of intensity is found ∼2.1–2.2 eV; this is the same energetic region as we have observed significant UV–vis spectral intensity and will be addressed in the correlation procedure presented in the following section. In short, this deficiency is expected as the CASCI/NEVPT2 approach is still limited by the feasible active space side, precluding the inclusion of adequate ligand orbitals that would allow for calculation of charge-transfer transitions.

Like the HS spectra, the computed LS spectra are also dominated by Δ*S* = 0, 1 transitions, primarily ^1^T and ^3^T excited states with some contributions from ^1^E and ^3^E states (Figures 7 and S12–S14). The low-energy region displays two features arising from the 1-^3^T_1_, 1-^3^T_2_, and 1-^1^T_1_ states (10,000–16,100 cm^–1^). Interestingly, inclusion of only σ-bonding interactions ((16,10) active space) leads to significant decreases in the 1-^3^T_1_ and 1-^3^T_2_ states, suggesting a significantly weaker LF when using this active space for the LS state. Also like the HS spectra, we observe better agreement with higher excitation energies. This discrepancy is primarily due to a major overestimation of contributions from the 3-^3^T_2_ and 1-^1^E states around 4.2 eV (34,000 cm^–1^).

### Correlation of Valence- and Core-Level Spectroscopies

Multiplet-rich metal ions such as Fe^2+^ provide extreme examples of dense LF manifolds with few spin-allowed transitions. Unlike optical spectroscopies, 2p3d RIXS allows these spin-forbidden final states to be readily accessed; however, these manifolds are often so *so* rich that the interpreting spectra can be difficult, particularly when CT transitions are also present. However, by combining multiple complementing spectroscopic methods to *selectively* access different manifolds, we can greatly enhance our ability to extract useful LF information. To demonstrate this, we have employed a global deconvolution procedure across a 0–32000 cm^–1^ (0–4 eV) energy range to combine the information content across the three spectroscopic approaches applied above.

**Figure 8:**
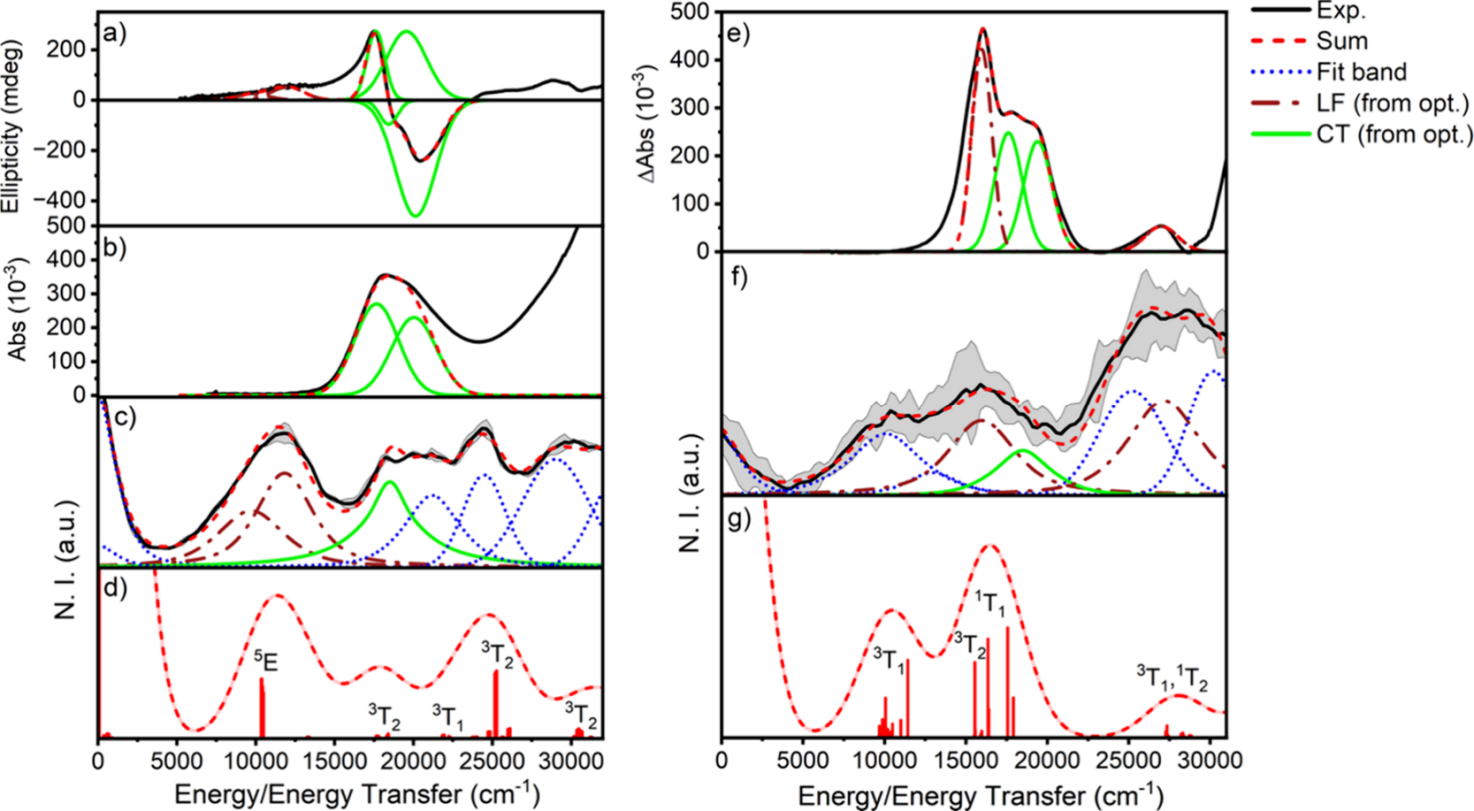
Example of globally fit spectra for HS (left, a–d) and LS (right, e–g) states together with representative examples of respective ab initio*-*calculated RIXS spectra. Spectra presented include (a) LIESST-induced HS 5*K*/5T MCD, (b) 220 K UV–vis, (c) 220 K 2p3d RIXS with a 709.25 eV incident energy, (d) corresponding HS CASCI/NEVPT2(22,13) RIXS spectrum (709.95 eV cut), (e) temperature-dependent UV–vis difference spectrum (220–80 K), (f) 50 K 2p3d RIXS with a 708.25 eV incident energy, and g) corresponding LS CASCI/NEVPT2(22,13) RIXS spectrum (708.8 eV cut). LF bands observed in both RIXS and optical spectra are shown in dark red (dashed dots), while CT bands are shown in green (solid). Additional fit bands based on fitting the RIXS spectra are shown in blue (dot). Standard deviations (σ) for each spectrum are provided as a shaded gray area.

1. We start by considering the temperature-dependent changes observed by UV–vis (inset of Figure 3) when moving from 220 to 80 K. These changes correspond to electronic states accessible under electronic absorption selection rules (Δ*S* = 0, Δ*l* = ± 1) that are significantly influenced with the SCO transition, i.e., MLCT/LMCT and LF states. This difference spectrum (80–220 K) can be reasonably fit using four bands centered at energies 15,970, 17,100, 19,500, and 27,200 cm^–1^ (Figure 8e).
2. Of the four bands fit in step 1), two are still prominently observed at 220 K (17,100 and 19,500 cm^–1^, Figure 8b), while two are only found upon decreasing temperature (15,970 and 27,200 cm^–1^). Additionally, no *negative* changes are observed when decreasing the temperature, suggesting that there are no bands *unique* to the HS state (e.g., the ^5^T_2_ → ^5^E LF transition) observed by UV–vis. Therefore, we assign the bands at 17,100 and 19,500 cm^–1^ as CT-type transitions and refer to these as 1-CT and 2-CT, respectively.
3. The assignments of 1-CT and 2-CT are reinforced by simultaneous deconvolution of the dominant components of the LIESST-induced HS MCD spectra (Figure 8a). These bands are fit as a pair of *pseudo A*-terms, indicating degeneracy in either the ground or excited states, each requiring a pair of oppositely signed bands split by approximately the SOC constant of Fe^II^ (∼640 cm^–1^).^70^
4. With 1-CT and 2-CT firmly assigned, we next consider the HS RIXS spectra (Figures 8c and S14) by globally fitting a minimum number of pseudo-Voigtian bands for all RIXS spectra obtained at different incident energies. Initially, bands 1-CT and 2-CT were fit with fixed energies based on UV–vis and MCD results. However, due to the broader nature of the RIXS spectra, 1-CT and 2-CT (17,100 and 19,500 cm^–1^) could be readily reduced to a single pseudo-Voigtian band centered at 18,500 cm^–1^. In addition to the CT band, two bands are required to fit the lowest-energy feature (9700 and 11,800 cm^–1^), which also correlate with a weak MCD intensity observed at ∼11,500 cm^–1^. To higher energy, three additional bands are fit at 22,000, 24,500, and 29,000 cm^–1^.
5. Moving to the LS RIXS manifold, we again treat CT-1 and CT-2 with a single CT band with a fixed energy of 18,500 cm^–1^. Additionally, fixed energy bands corresponding to the LS-dependent UV–vis features at 15,970 and 27,200 cm^–1^ were included. In addition to these three bands, two additional lower energy (10,200 and 13,100 cm^–1^) and higher energy (24,800 and 29,900 cm^–1^) bands were included in our minimal global fitting procedure across RIXS spectra collected at all presented incident energies (Figures 8f and S15).

Plots of the global spectral band deconvolution for all RIXS cuts are provided in Figures S14 and S15. Using the minimal band set summarized in Table 4, we can further draw comparisons with our calculations to propose several LF state assignments. First, we reiterate that our calculations support significant RIXS intensity only for Δ*S* = 0, 1 transitions (rather than Δ*S* = 2). For the HS state, the spin-allowed 1-^5^E state is calculated to dominate the low-energy region, with only very minor contributions from 1-^3^T_1_ (Figures S8–S11). Additionally, there are no observed CT states in this low energetic range; therefore, we assign both bands 1 and 2 of the HS state to the ^5^E state, corresponding to the sole spin-allowed transition. This assignment is also supported by the observation of MCD-based intensity in this energetic region, and is energetically consistent with the ^5^T → ^5^E transition for HS octahedral Fe^II^ observed for other SCO complexes.^63,71^ Moving up in energy, the 1-^3^T_2_ state is calculated to significantly contribute to the RIXS around 18,000 cm^–1^; however, the CT transition masks this energetic region, precluding a conclusive assignment. The following three bands (4, 5, 6) are reasonably well-separated and match energetically with the following three triplet final states, and given the lack of significant CT intensity in this region (from UV–vis and MCD), we assigned these as 2-^3^T_1_, 2-^3^T_2_, and 3-^3^T_2_ states.

**Table 4:** Summaries of Bands Resulting from HS and LS Global Fitting Procedures together with Proposed State Assignments and Corresponding CASCI/NEVPT2 Calculated Energies^a^

	energy		state	active space					
				(12,8)		(16,10)		(22,13)	
	eV	cm^–1^		eV	cm^–1^	eV	cm^–1^	eV	cm^–1^
HS band									
elastic	0	0	1-^5^T_2_	0	0	0	0	0	0
1	1.2	9700	**1-**^**5**^**E**	1.41	11,390	1.32	10,620	1.32	10,660
2	1.5	11,800							
3	2.3	18,500	CT						
4	2.7	22,000	2-^3^T_1_	2.71	21,890	2.70	21,790	2.69	21,710
5	3.0	24,500	2-^3^T_2_	2.98	24,020	2.99	24,110	2.98	24,030
6	3.6	29,000	3-^3^T_2_	3.72	30,020	3.67	29,620	3.66	29,560
LS band									
elastic	0	0	1-^1^A_1_	0	0	0	0	0	0
1	1.3	10,200	1-^3^T_1_	1.05	8,430	0.84	6,780	1.25	10,040
2	1.6	13,100	1-^3^T_2_	1.83	14,740	1.58	12,770	1.98	15,960
3	2.0	15,970	**1-**^**1**^**T**_**1**_	2.20	17,770	2.01	16,220	2.19	17,680
4	2.3	18,500	CT						
5	3.1	24,800	2-^3^T_1_	3.22	25,940	2.82	22,750	3.39	27,330
6	3.4	27,200	**1-**^**1**^**T**_**2**_	3.63	29,250	3.32	26,820	3.54	28,530
7	3.7	29,900	1-^3^E	3.65	29,510	3.25	26,210	3.82	30,790
			3-^3^T_1_	3.68	29,700	3.27	26,410	3.88	31,300

a States resulting from spin-allowed (Δ*S* = 0) transitions are highlighted in bold.

Similar to the HS state, no significant CT contributions are observed below 16,000 cm^–1^ in the LS UV–vis spectrum (80 K, Figures 2 and 8). Therefore, the RIXS spectral intensity to lower energies must arise from LF transitions. Two bands are fit in this region, and similarly calculations display two states, 1-^3^T_1_ and 1-^3^T_2_ (Table 4 and Figures S12–S14). While both show significant energy dependence on active space composition, the distinct lack of alternative possibilities allows us to assign bands 1 and 2 as 1-^3^T_1_ and 1-^3^T_2_, respectively. The appearance of band 3 in the UV–vis and its complete dependence on temperature allow this feature to readily be assigned to the spin-allowed 1-^1^T_1_ state. Likewise, the complete temperature dependence of the UV–vis band at 27,200 cm^–1^ allows assignment of this feature (band 6) to the spin-allowed 1-^1^T_2_ state. Similar to the HS state, UV–vis spectra do not indicate another strong CT transition in the 20,000–30,000 cm^–1^ regime, and we therefore assign band 5 to the 2-^3^T_1_. Band 7 is energetically consistent with either the 1-^3^E or 3-^3^T_1_ (or both), precluding a definitive assignment.

## Discussion

Herein, we have employed a multidimensional approach to characterize the LF and magnetic properties of SCO complex **1** using valence, L_3_-edge XAS, and 2p3d RIXS spectroscopies together with ab initio theory at the CASCI/NEVPT2 level. In particular, we have demonstrated how these methods can be used to complement one another and enhance the certainty of LF state assignments in both the HS and LS states of Fe^II^. Our temperature-dependent UV–vis/nIR and MCD measurements allowed for the determination of the Δ*S* = 0 LF excited state within the accessible energy window (6000–30,000 cm^–1^). Furthermore, the dependence of MCD intensity on the spin-angular momentum implicated significant Fe^II^ character in the absorption features ranging from ∼16,000 to 22,000 cm^–1^, supporting the presence of a ligand/metal CT transition in this energetic feature. Meanwhile, the 2p3d RIXS accesses a significantly wider energy range, where the combination of Δ*S* = 0, 1 excited states (as supported by CASCI/NEVPT2 calculations) as well as CT transitions with significant metal character produces extremely rich spectra that can be challenging to interpret. In this way, UV–vis/nIR and MCD provide critical, complementing information regarding Δ*S* = 0 and CT transition contributions to the 2p3d RIXS, allowing for a more accurate interpretation.

As we have seen presently, the LIESST/SOXIESST behavior of some SCO complexes becomes critically important to account for when probing electronic structure and can serve as a double-edged sword. On the one hand, this behavior can present a challenge when probing the LS state using techniques that can generate a significant number of photons in the UV–vis/nIR region. Simultaneously, below *T*_c,LIESST_, this property has allowed us to employ spin-sensitive techniques relying on the differential population of the magnetic ground state manifold–in particular, this has allowed for the novel employment of low-temperature MCD to probe the spin-allowed ^5^T → ^5^E LF transition of the HS state. Importantly, a comparison of our Fe 2p3d RIXS collected at both 15 and 220 K support that the electronic structure of the HS state is the same whether accessed via SOXIESST or thermally (Figure S8).

Beyond complementing valence- and core-level spectroscopic measurements, ab initio theory has served as a critical support in the formal assignment of spectroscopic terms with their corresponding LF excited states. While CASSCF/NEVPT2 is an effective approach to providing insight into the valence electronic states of transition metals, this method is not readily employed to the prediction of intensity contributions in high-energy electron-in/electron-out spectroscopies such as 2p3d RIXS. These difficulties arise as inclusion of the 2p orbitals in the active space is required to calculate the 2p3d RIXS intermediate states, and the convergence of the resulting wave function is poorly behaved when including orbitals near or at 100% occupancy in the active space. The CASCI/NEVPT2 approach employs a compromise, wherein an active space containing the necessary valence orbitals are fully optimized prior to the inclusion of the 2p orbitals. As a result, this method provides an energetically relaxed valence active space, while allowing for the calculation of core-to-valence (and valence-to-core) electronic transitions. Generally, we have demonstrated that this method is effective in generating excellent LF state energies with reasonable intensities for the RIXS process, particularly in the case of the HS state of **1**. We note that these intensities appear to become less reliable at higher energies where there is a significantly greater overlap of LF terms and where overlap with CT-type transitions occurs. In particular, the calculated intensity of the 3-^3^T_2_ terms is significantly overestimated for the LS state, while that of the 3-^3^T_1_ is underestimated. Additionally, CT-type transitions are not reproduced as the number of orbitals and electrons required in the active space to do so generally makes these calculations forbiddingly expensive. However, the energetic positions and RIXS intensities of the majority of calculated LF states appear reasonable enough that spectral regions arising from CT transitions are highlighted by a lack of calculated intensity, particularly when taken together with the CT energies taken from our optical spectroscopic measurements.

By bringing together our experimental LF state assignments from the combined experimental optical + RIXS approach with those obtained from the CASCI/NEVPT2 theory, we assigned the LF states accessible in the 0–32,000 cm^–1^ (0–4 eV) energetic region. Using these state assignments, we can further fit LF parameters of both the HS and LS states of **1** using the angular overlap model as described in the SI; these results are summarized in Table 5.

**Table 5:** Summary of Experimental and Calculated LF Parameters of Compound **1**

state		LF parameters						
		(cm^–1^)						
		10*D*_q_	*B*	*C*	*e*_σ_	*e*_π_	*B/B*_*0*_^a^	*C/B*
HS	exp.	10,570	920	3820	3860	680	0.94	4.16
	(6,5)CASSCF/NEVPT2	10,580	1270	3210	3980	680	1.30	2.54
	(16,10)CASSCF/NEVPT2	11,800	1070	3420	4620	1030	1.10	3.20
	(12,8)CASCI/NEVPT2	10,990	1190	3600	4240	860	1.22	3.03
	(16,10)CASCI/NEVPT2	10,400	1160	3670	3820	710	1.20	3.16
	(22,13)CASCI/NEVPT2	10,100	1170	3640	3850	720	1.20	3.11
LS	exp.	18,250	960	3230	6230	220	0.99	3.36
	(6,5)CASSCF/NEVPT2	20,350	1,020	3980	6780	0	1.06	3.88
	(16,10)CASSCF/NEVPT2	20,680	840	4120	6890	0	0.86	4.91
	(12,8)CASCI/NEVPT2	19,540	1,110	4000	6510	0	1.14	3.61
	(16,10)CASCI/NEVPT2	17,950	1060	4030	5980	0	1.09	3.80
	(22,13)CASCI/NEVPT2	19,180	990	3270	6390	0	1.02	3.29

a *B*/*B*_0_ calculated using the free ion value of *B*_0_ = 971 cm^–1^ for Fe^II^.^72^

Generally, the LF splitting 10*D*_q_ (defined for the present system as 3*e*_σ_ - 2*e*_π_) of the HS state is approximately half that of the LS state. Among the experimentally determined values, only a minor decrease in the interelectronic repulsion Racah parameter *B* is observed between the HS and LS states, indicating a similar level of covalency. This is consistent with the near constant sum of N and Fe electron densities during photoinduced SCO, as observed by N K-edge measurements.^73^ This trend is consistent with our calculations as well. Furthermore, *e*_σ_ increases significantly in the LS state, a reflection of the near-symmetric contraction of the metal–ligand bonds. Despite providing a relatively small contribution to the HS LF splitting, *e*_π_ is reduced in the LS state and found to be zero for all calculations. The observation of underlying asymmetry in these systems is consistent with the proposal that nontotally symmetric vibrational modes contribute to the mediation of intersystem crossing for other SCO complexes, such as [Fe(bpy)]^2+^.^10,74,75^

Comparing the ability of our calculations to match the experimentally determined LF parameters, we find that the CASCI/NEVPT2 calculations generally provide better agreement in terms of 10*D*_q_ but underestimate the metal–ligand covalency as reflected by overestimates in *B*/*B*_0_. This deviation is more pronounced for the smaller (12,8) and (16,10) CASCI active spaces. The use of a larger active space including the full bonding orbital complement [(16,10) for CASSCF/NEVPT2, (22,13) for CASCI/NEVPT2] generally provides better estimates for both 10Dq and *B*. However, we note that our smallest active spaces, including only the 3*d*-centered antibonding orbitals (both with and without the 2p orbitals), still appear to provide a reasonable approximation.

Several previous studies have also explored the electronic excited-state manifold and LF splitting properties of SCO complexes. For example, Decurtins et al. reported the optically accessible electronic state of [Fe(ptz)_6_](BF_4_)_2_, where similar excited-state energies to the presently investigated **1** complex for the ^5^E (12,250 cm^–1^) of the HS state and ^1^T_1_ and ^1^T_2_ (18,400 and 26,650 cm^–1^) of the LS state were reported.^62^ Unlike the present study, a significantly smaller *B* (600 cm^–1^) was reported for the LS state than that estimated for the HS state (760–1058 cm^–1^). However, the limited number of observable electronic transitions in these cases precludes the ability to accurately determine the LF parameters. Methods of both observing and accurately assigning a broader manifold of LF states, as we have demonstrated presently, may prove critical in determining the influence of interelectronic repulsion in the SCO phenomenon. In fact, the similar metal–ligand covalency strength as indicated by the simulated interelectronic repulsion Racah parameters seems to be of paramount importance for stabilizing the different forms of the SCO systems. However, we emphasize that studying the dynamic SCO phenomenon would require operando conditions and inclusion of excited-state dynamics in our calculations, which is beyond the scope of the present study. Hence, further dedicated studies are required before the role of such phenomenon can be fully understood in SCO systems.

## Conclusions

We have provided a detailed study of the electronic properties of SCO molecular complex **1** using a combination of complementing experimental (SQUID, absorption, MCD, and Fe 2p3d RIXS) and theoretical (CASSCF/NEVPT2 and CASCI/NEVPT2) methods. We have further examined the power of exploiting the LIESST/SOXIESST processes to investigate LF excited states, including the novel use of the LIESST effect to generate the photoinduced HS state for study using MCD while also highlighting the experimental challenges that can be expected when applying soft X-ray methods to SOXIESST-active SCO systems. Furthermore, we have demonstrated the capabilities of the current state-of-the art ab initio CASCI/NEVPT2 approach in calculating 2p3d RIXS and refining LF excited-state assignments. The methodology we have presented provides a robust, albeit labor intensive, approach to exploring the LF properties of SCO systems, which may be further applied to a wider range of systems to provide a more systematic understanding of the influence of metal–ligand bonding interactions and covalency in imparting SCO behavior. Developments are underway in our laboratories toward expanding the size of active spaces that can be treated at the CASCI/NEVPT2 level with the aid of approximate CI theories and to be able to compute valence and core excited states at the CASCI/NEVPT2 level including spin-vibronic coupling effects.^76−78^

## Data Availability

All graphed data presented in the manuscript and Supporting Information can be found free of charge in ascii format in the Edmond Open Research Repository (https://doi.org/10.17617/3.G5Y6HF).

## Supporting Information Available

Additional experimental details, including a description of geometric structure and angular overlap model considerations, as well as additional spectroscopic analysis for ^57^Fe Mössbauer spectroscopy, SQUID, MCD spectroscopy, Fe 2p3d RIXS spectroscopy, and CASCI/NEVPT2 and CASSCF/NEVPT2 calculations (PDF)
