# Supplementary material for: Correlating Valence and 2p3d RIXS Spectroscopies: A Ligand-Field Study of Spin-Crossover Iron(II)
Source: Inorg Chem. 2024 Apr 8;63(16):7386–400. doi: 10.1021/acs.inorgchem.4c00435 (PMC11040727; doi:10.1021/acs.inorgchem.4c00435)
Supplement: Supplementary file 1 — ic4c00435_si_001.pdf [file ic4c00435_si_001.pdf]

Supplementary Information:

Correlating Valence and 2p3d RIXS Spectroscopies: A Ligand-Field Study of Spin-Crossover Iron(II)

Casey Van Stappen[a,°], Benjamin E. Van Kuiken[a,b], Max Mörtel[c], Kari O. Ruotsalainen,[d,∞] Dimitrios Maganas[e], Marat M. Khusniyarov[c], Serena DeBeer[a]*

[a]Max Planck Institute for Chemical Energy Conversion, Stiftstrasse 34-36, 45470 Mülheim an der Ruhr, Germany

email: serena.debeer@cec.mpg.de

[b]European XFEL, Holzkoppel 4, 22869 Schenefeld, Germany

[c]Department of Chemistry and Pharmacy, Friedrich-Alexander-Universität Erlangen-Nürnberg (FAU), Egerlandstrasse 1, 91058 Erlangen, Germany

[d]Synchrotron SOLEIL, L'Orme des Merisiers, Départementale 128, 91190 Saint-Aubin, France

[e]Max-Planck-Institut für Kohlenforschung. Kaiser-Wilhelm-Platz 1, 45470 Mülheim an der Ruhr, Germany

[°]Current address: University of Texas at Austin, Department of Chemistry, 105 E 24th St., Austin, TX 78712

[∞]Current address: Helmholtz-Zentrum Berlin für Materialen und Energie, Albert Einstein Straße 15, 12489, Berlin, Germany

Table of Contents

1. Geometric Structure and Angular Overlap Considerations **2**
2. ^{57}Fe Mössbauer **3**
3. SQUID **4**
4. MCD **5**
5. RIXS **7**
6. CASSCF/NEVPT2 & CASCI/NEVPT2 ..... **8**
7. Band Deconvolution Analysis **15**
References **18**

1. Geometric Structure and Angular Overlap Considerations

The $[Fe^{II}(H_2B(pz)_2)_2phen]^0$ complex (**1**) is *pseudo*-O_h in geometry, with a strict C_2 symmetry (Figure 1). All 6 N-ligands are sp^2 hybridized, being part of conjugated, delocalized rings. Therefore, all ligands participate in 1) σ-donating, and 2) π_{oop}-donating (oop = out-of-plane) interactions with Fe, while there are no significant π_{ip} interactions (ip = in-plane). As a result, although seemingly *pseudo*-O_h, a C_2 symmetric model is necessary to accurately simulate the electronic structure of this complex. In this model, the minimum descriptive geometry of **1**, ligating N can be divided into 3 types, and summarized by 9 angles (Scheme 1).

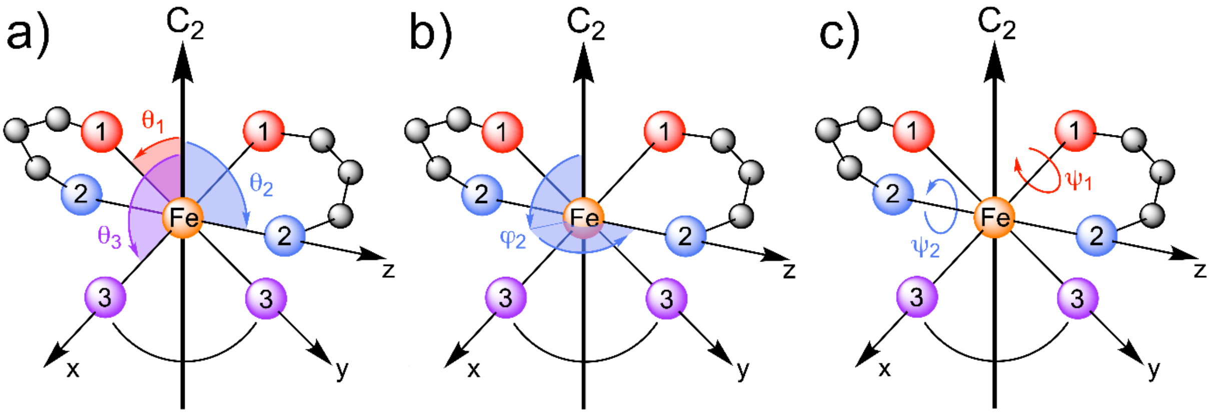

Scheme 1. Descriptive angles[a] of **1**. [a]Under C_2 symmetry, the coordination environment around Fe can be described in terms of 3 unique ligand atoms, each with a unique angle relative to the C_2 axis (labeled θ_n, n = 1-3, shown in a). Each of these ligands also interacts asymmetrically with the central Fe, requiring further distinction by twist angle, φ_n (b), and torsion angle, ψ_n (c). As $\varphi_3 = 0$, only φ_1 and φ_2 are shown. Axes *x, y, z* are provided along bonds in the *pseudo*-O_h coordinate frame.

The C_2 axis runs along the plane formed between atom groups 1 and 3, bisecting the 1-Fe-1 and 3-Fe-3 angles. θ is defined as the bond angle from the C_2 axis. φ is the twist angle, defined by the angle between the C_2 axis, a point along the intersection of the x,y plane and a plane orthogonal to the C_2 axis containing the ligand atom of interest, and the atom of interest. Lastly, ψ is the torsion angle between the C_2 axis, Fe, the atom of interest, and bound neighboring atom (shown as gray spheres in Scheme 1). When comparing the HS and LS structures, we see that in addition to the symmetric contraction of all bond distances by ~0.19 Å, small changes in bonding angles also occur. In the HS structure of **1**: θ_1 = 45.3°, θ_2 = 88.6°, θ_3 = 142.5°; $\varphi_1 = \varphi_3$ = 0°, φ_2 = 88.6°; ψ_1 = 96.8°, ψ_2 = 53.1°, ψ_3 = 0°. In the LS structure, these angles are: θ_1 = 42.8°, θ_2 = 90.5°. θ_3 = 138.8°; $\varphi_1 = \varphi_3$ = 0°, φ_2 = 90.5°; ψ_1 = 91.3, ψ_2 = -51.7°, ψ_3 = 0°.

To determine the ligand field parameters of **1**, an O_h approximation was made by using $\theta_{1,2,3}$ = 45°, 90°, 135°; $\varphi_{1,2,3}$ = 0°, 90°, 0°; $\psi_{1,2,3}$ = 90°, 45°, 0°. While each of the six coordinating atoms of Fe can each interact through a sigma interaction with the d_{x2-y2} and d_{z2} orbitals, calculated as 6 σ-interactions/2 orbitals = 3σ. Meanwhile, significant π-bonding interactions between Fe and the surrounding ligands will only form between the out-of-plane π-orbitals of the $H_2B(pz)$ and phen ligands and the d_{xy}, d_{xz}, and d_{yz} ligands, providing 6 π-interactions/3 orbitals = 2π. Therefore, the ligand field splitting parameter of **1** is defined as $10D_q = 3e_\sigma - 2e_\pi$. Using this definition together with the assigned LF states in Table 4 of the main text, AOMX was used to fit parameters $10D_q$, e_σ, and e_π, along with Racah parameters B and C.

2. ^{57}Fe Mössbauer

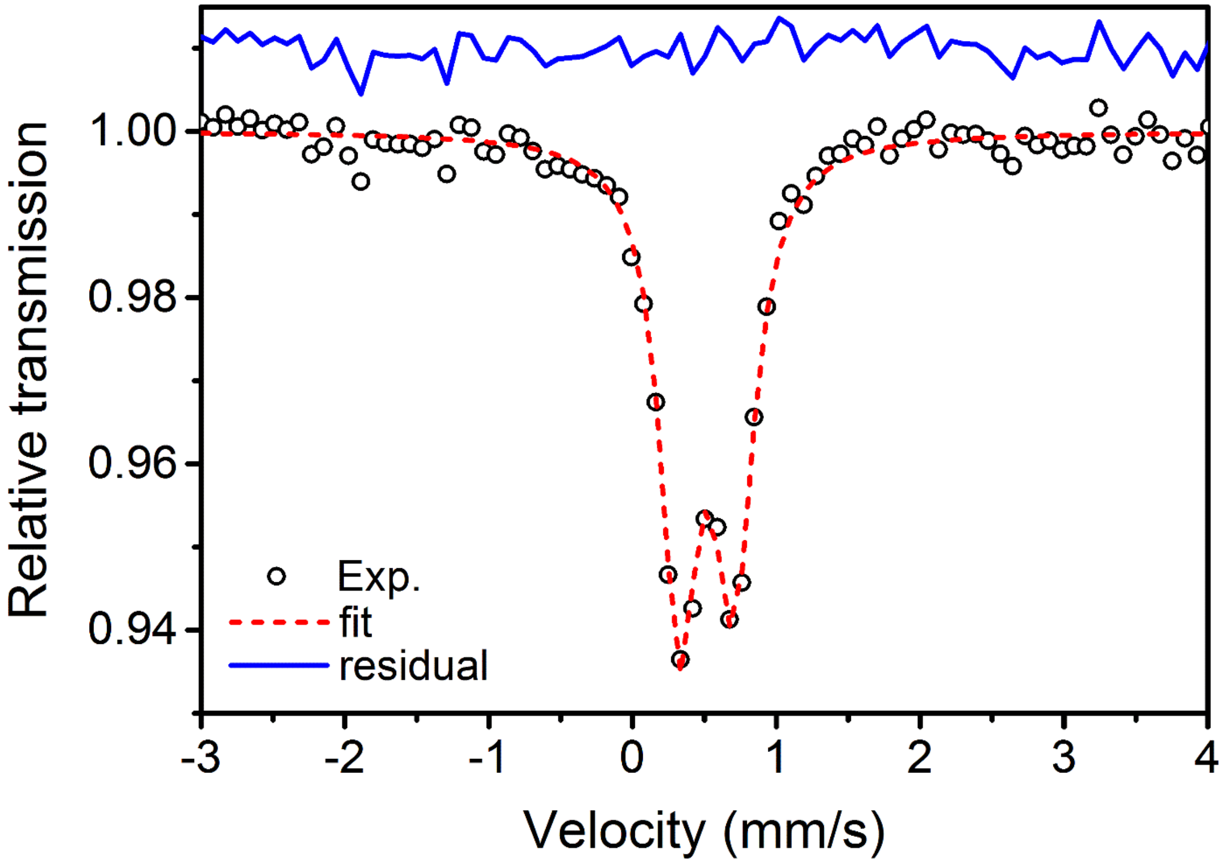

Figure S1. ^{57}Fe Mössbauer spectrum (1.6 K, 0 T) of **1** prepared as a polysiloxane mull (circles, black) along with corresponding fit (red, dashed) using a single doublet with δ = 0.51 mm/s, ΔE_Q = 0.38 mm/s, and an asymmetric linewidth of 0.31 mm/s (1.09 asymmetry factor). Residual shown above in blue, solid. These results are consistent with previously published data of LS **1**.[1]

3. SQUID

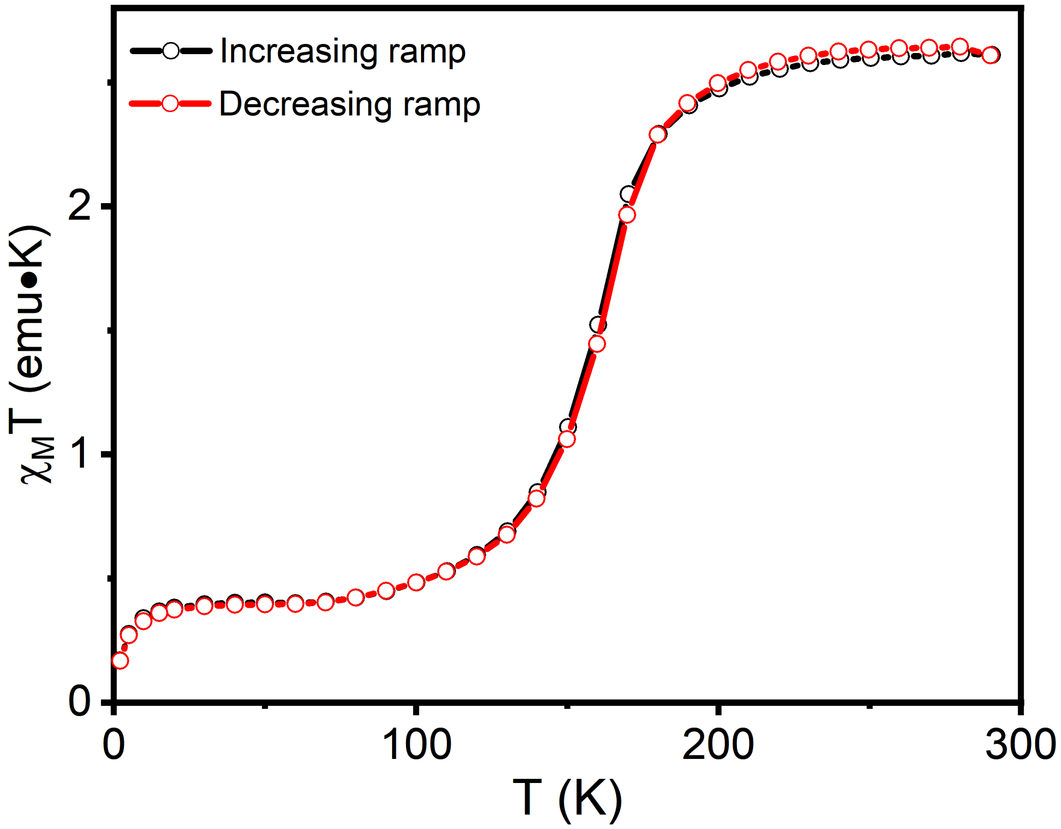

Figure S2. Magnetization measurements of the **1** complex sweeping up (black) in temperature, and back down in temperature (red) at 1 T. Hysteresis consistent with previous reports is observed.[2]

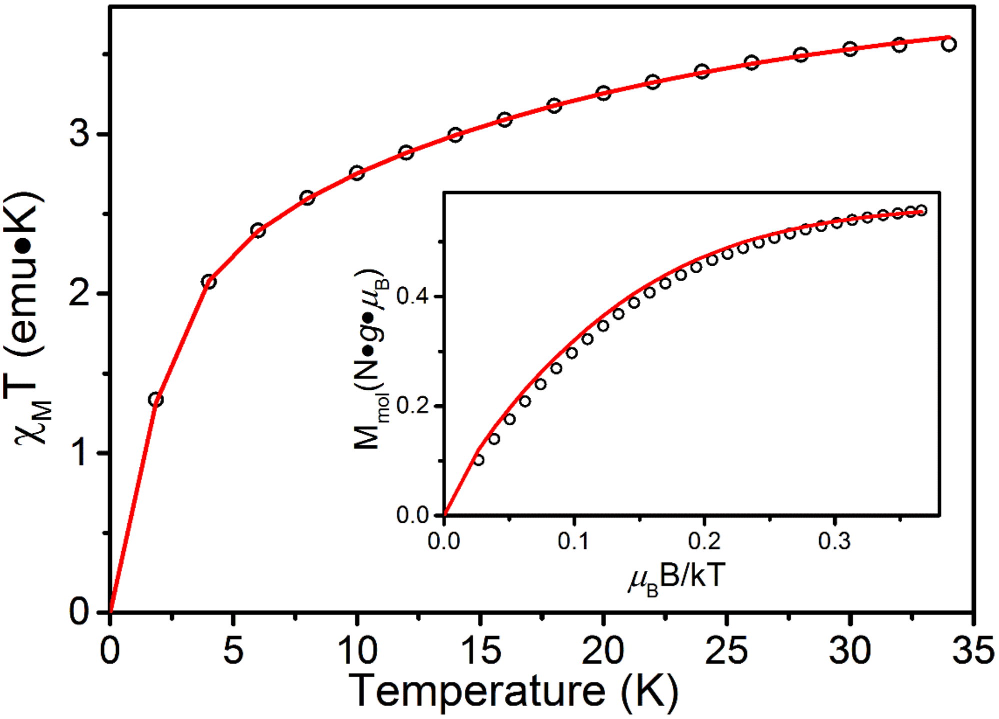

Figure S3. Variable-temperature dc SQUID measurements performed at 1 T of **1** in the LIESST-induced HS state. Higher fields resulted in sample torquing, and the limited sample volume precluded the use of an affixing agent. Corresponding fits (summarized in Table 1 of the main text) are shown (red, solid).

4. MCD

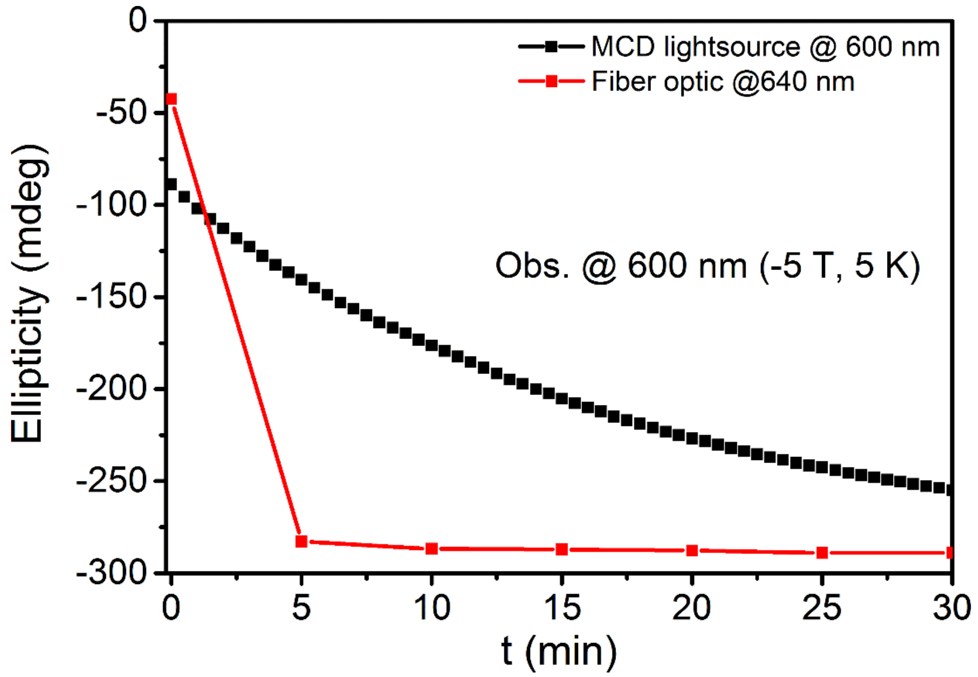

Figure S4. MCD signal dependence on sample irradiation by fiber optic (red) and the MCD light source itself (black). Measurements were performed at -5 T, 5 K.

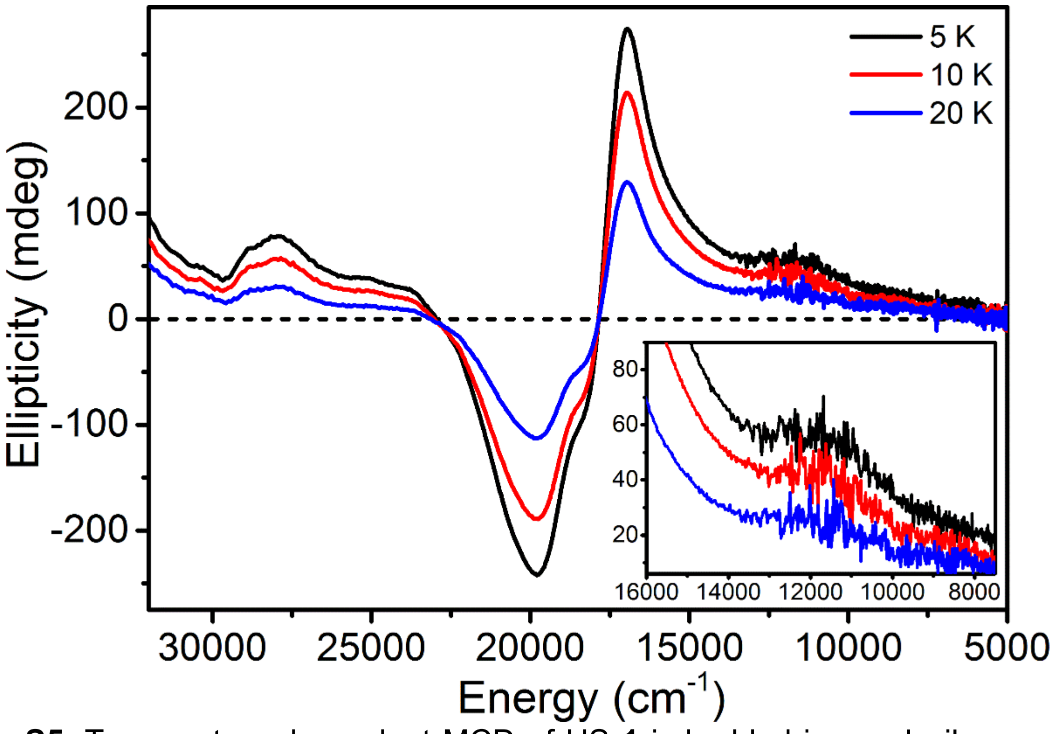

Figure S5. Temperature dependent MCD of HS **1** imbedded in a polysiloxane mull. Spectra were collected at an applied field of 5 T. The high-spin state was generated by exposure of the sample to light irradiation (640 nm) until signal intensity at 590 nm was saturated. Re-exposure to light irradiation was performed between individual scans of field and temperature. Poorer S/N is observed in the 8000-13000 cm^{-1} region as this constitutes the overlap of the respective very low and very high energy regimes of the PMT and InGaAs detectors employed.

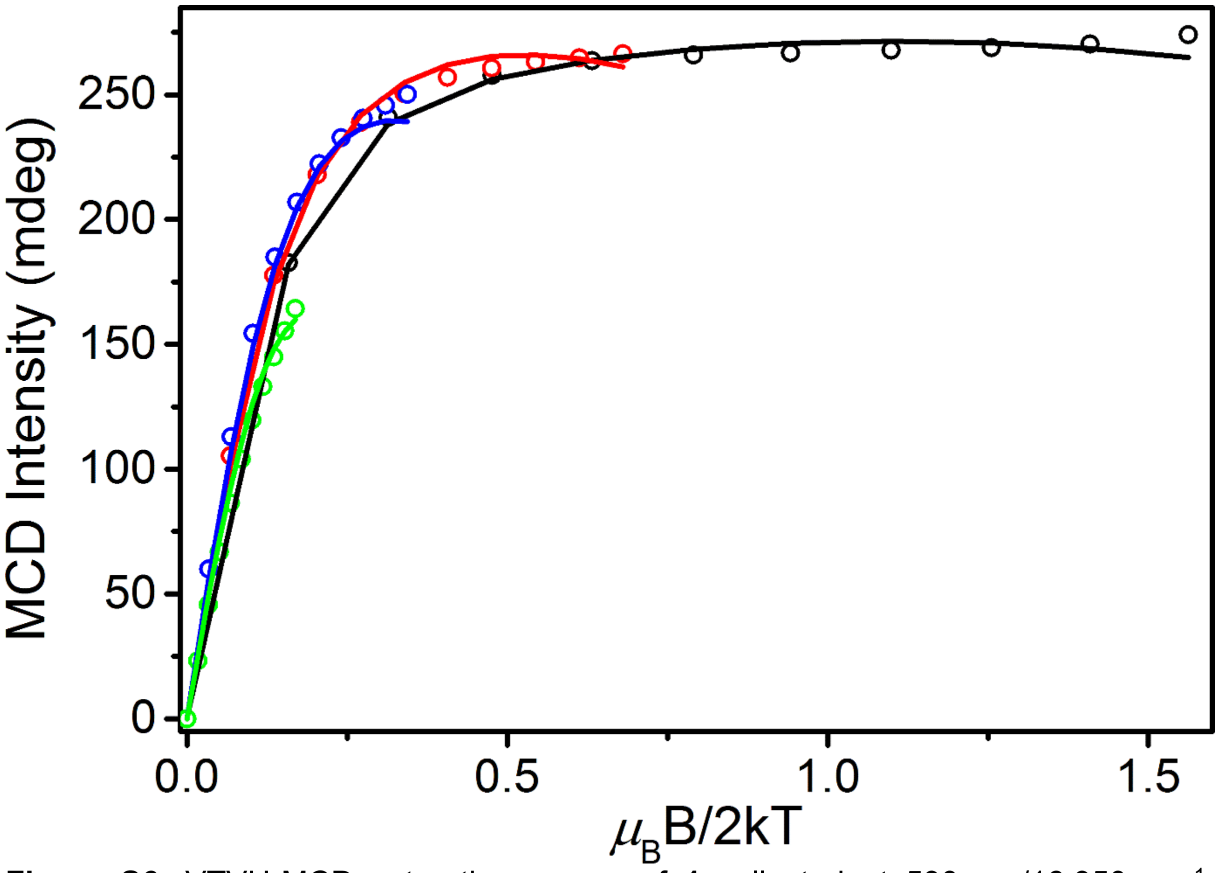

Figure S6. VTVH-MCD saturation curves of **1** collected at 590 nm/16,950 cm^{-1}. Experimental data are provided as circles, and the corresponding fit as solid lines. Spectra were collected over a range of 0-10 T at temperatures of 2 K (black), 5 K (red), 10 K (blue), and 20 K (green). Corresponding ZFS fitting parameters are summarized in the main text, Table 1.

It has previously been observed that LIESST can occur in both directions (converting LS to HS, and HS back to LS) depending on the energy of the incident radiation utilized.[3] While irradiation of the LS state in the energetic range of the $^3T_1$ and $^1T_2$ states (typically at 500-700 nm) induces trapping of the HS state at low temperature, irradiation in the range of the $^3T_1$ and ^5E (800-1000 nm) of the HS state has been also shown to induce reformation of the LS state.[3] MCD measurements of **1** prior to irradiation at 5 K, 5 T still exhibit considerable spectral intensity, despite not containing any trapped HS complex as demonstrated via ^{57}Fe Mössbauer, Figure S1. Further monitoring of MCD signal intensity as a function of time exposed to the MCD light source itself at 640 nm demonstrated that the incident beam was sufficiently intense to induce LIESST (Figure S5). Therefore, it is possible that the intensity of the ~11,700 cm^{-1} feature, assigned to the $^5T \rightarrow {}^5E$ transition, is partially diminished by the probing radiation of the MCD spectrometer.

5. RIXS

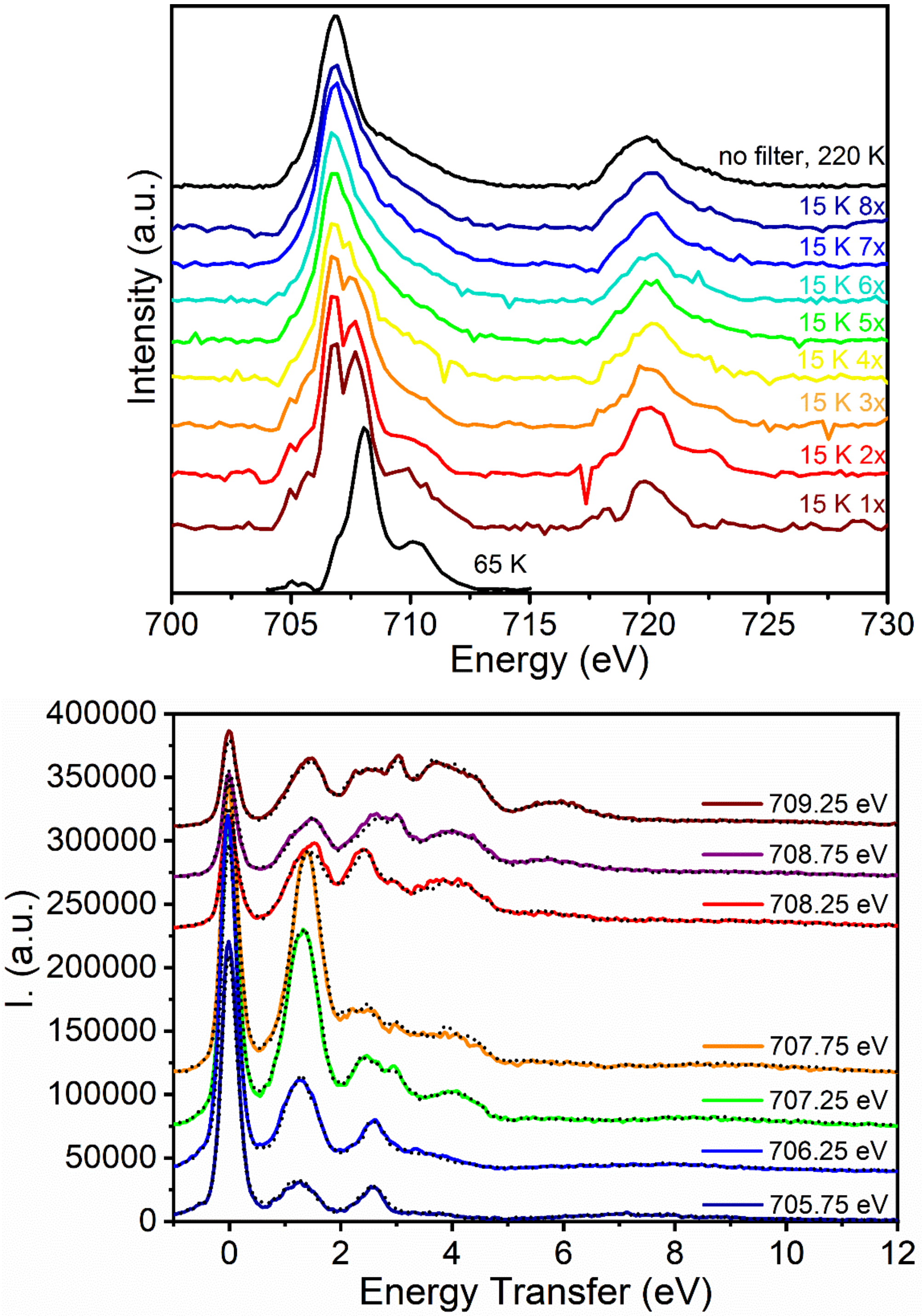

Figure S7. (top) Irradiation-dependent Fe $L_{2,3}$-edge spectrum of **1**. Spectra indicated by *N*x were collected on a single sample spot *N* number of times. The top spectrum is the pure HS spectrum collected at 220 K, and the pure LS spectrum collect at 65 K with additional Co filters is shown on bottom. (bottom) Comparison of RIXS collected at 220 K (solid lines) with that collected at 15 K under equivalent scanning/beam attenuation conditions (dotted lines).

6. CASSCF/NEVPT2 & CASCI/NEVPT2

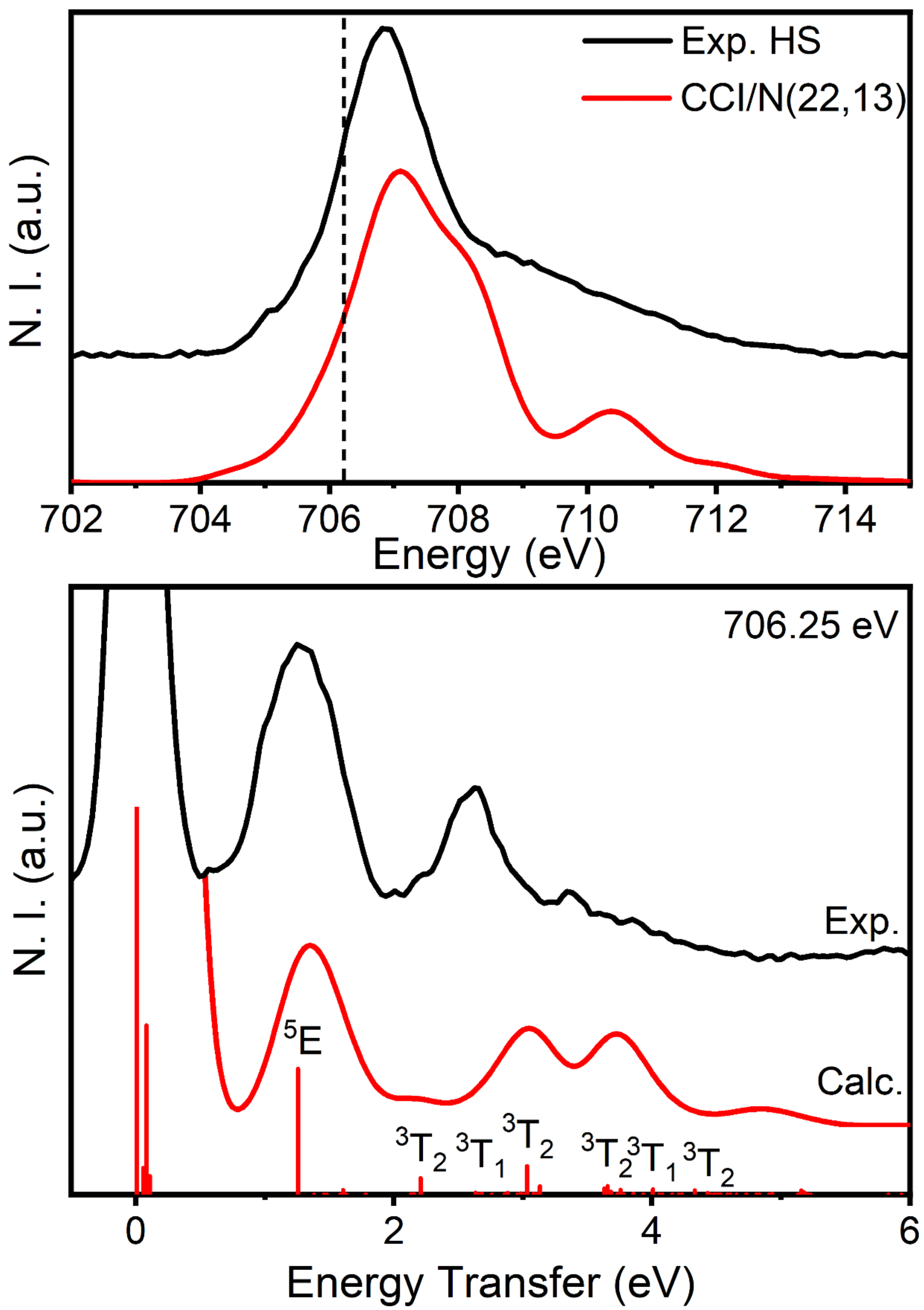

Figure S8. (top) Fe L_3-edge of HS **1** (black) compared with that calculated at the SA-CASCI/NEVPT2(22,13) level (red). An energy shift of -0.7 eV is applied to the calculated spectrum. A dashed vertical line indicates the incident energy used to collect the spectra in the bottom graph. (bottom) Experimental (black) vs. SA-CASCI/NEVPT2(22,13) calculated (red) Fe 2p3d RIXS at an incidence energy of 706.25 eV. The calculated Fe 2p3d RIXS represents the calculated spectrum at a 706.95 eV incident energy.

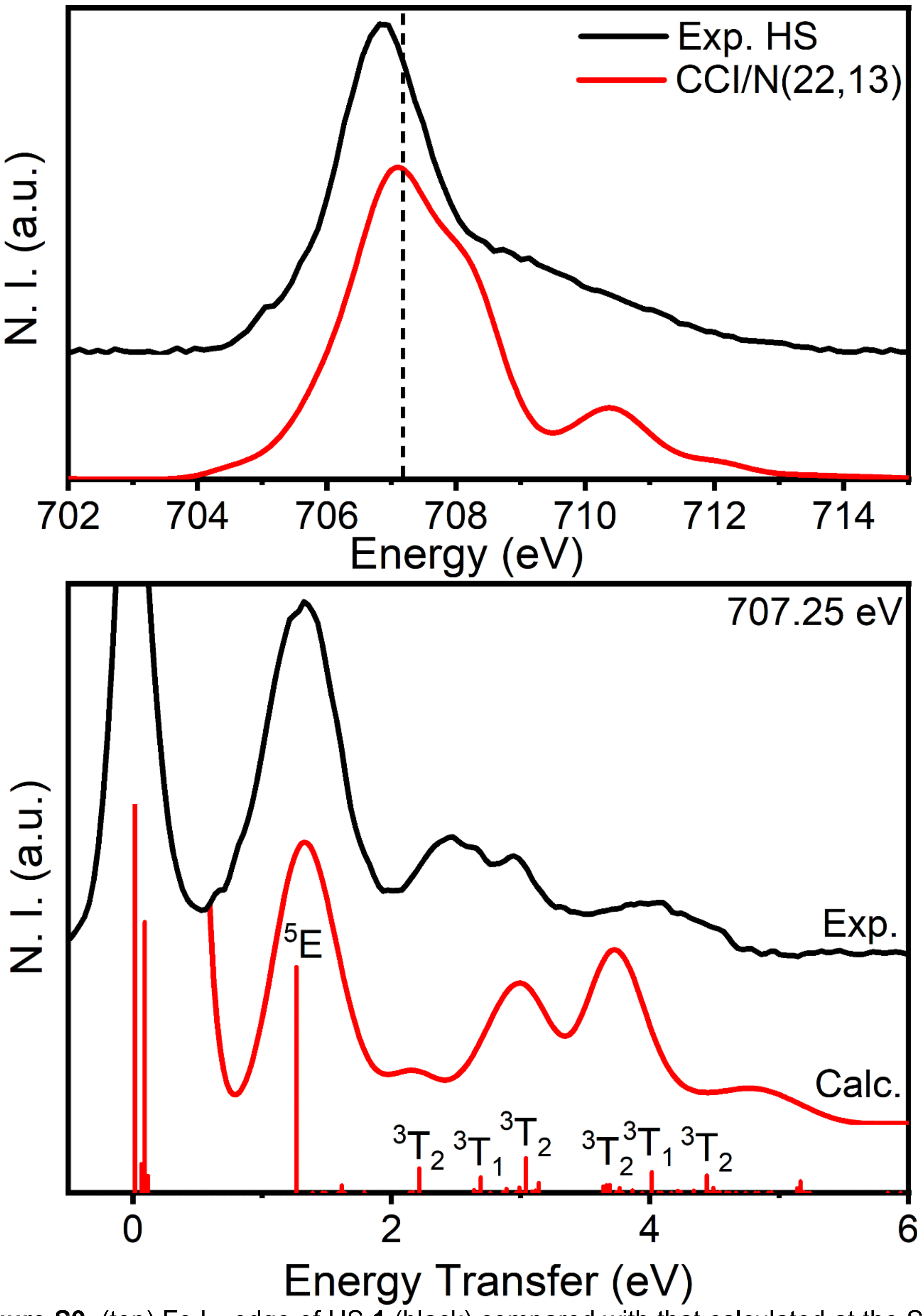

Figure S9. (top) Fe L_3-edge of HS **1** (black) compared with that calculated at the SA-CASCI/NEVPT2(22,13) level (red). An energy shift of -0.7 eV is applied to the calculated spectrum. A dashed vertical line indicates the incident energy used to collect the spectra in the bottom graph. (bottom) Experimental (black) vs. SA-CASCI/NEVPT2(22,13) calculated (red) Fe 2p3d RIXS at an incidence energy of 707.25 eV. The calculated Fe 2p3d RIXS represents the calculated spectrum at a 707.95 eV incident energy.

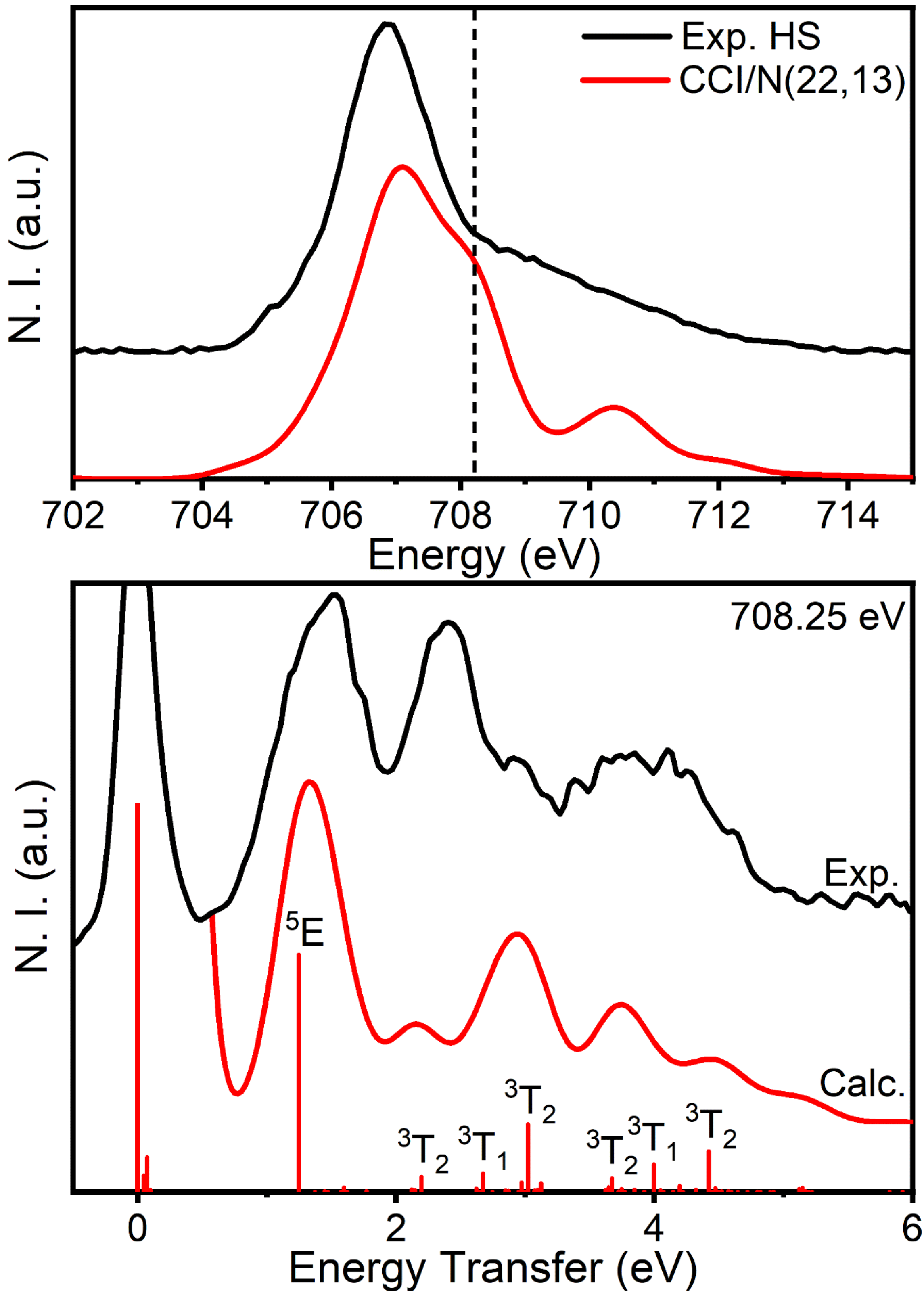

Figure S10. (top) Fe L_3-edge of HS **1** (black) compared with that calculated at the SA-CASCI/NEVPT2(22,13) level (red). An energy shift of -0.7 eV is applied to the calculated spectrum. A dashed vertical line indicates the incident energy used to collect the spectra in the bottom graph. (bottom) Experimental (black) vs. SA-CASCI/NEVPT2(22,13) calculated (red) Fe 2p3d RIXS at an incidence energy of 708.25 eV. The calculated Fe 2p3d RIXS represents the calculated spectrum at a 708.95 eV incident energy.

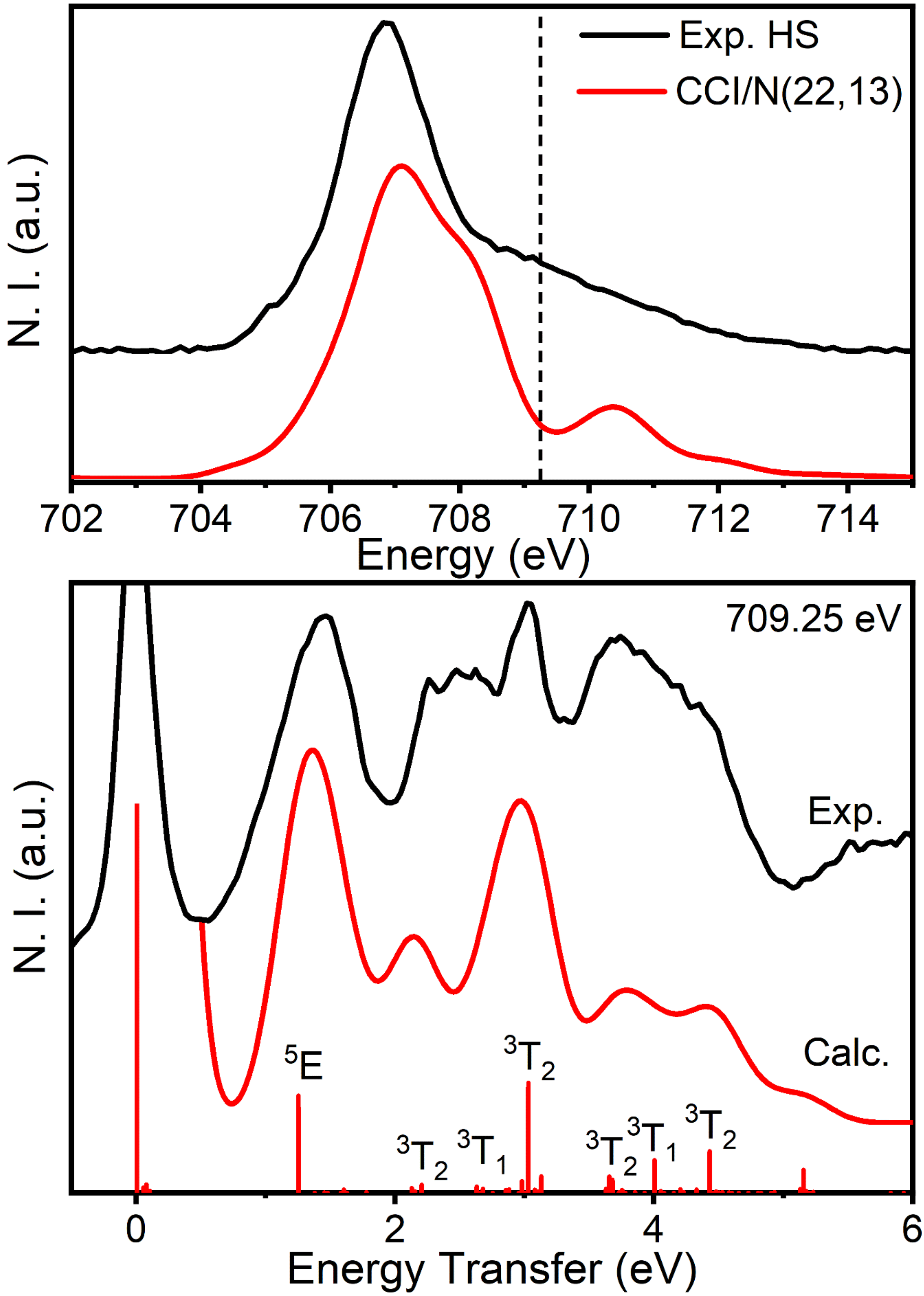

Figure S11. (top) Fe L_3-edge of HS **1** (black) compared with that calculated at the SA-CASCI/NEVPT2(22,13) level (red). An energy shift of -0.7 eV is applied to the calculated spectrum. A dashed vertical line indicates the incident energy used to collect the spectra in the bottom graph. (bottom) Experimental (black) vs. SA-CASCI/NEVPT2(22,13) calculated (red) Fe 2p3d RIXS at an incidence energy of 709.25 eV. The calculated Fe 2p3d RIXS represents the calculated spectrum at a 709.95 eV incident energy.

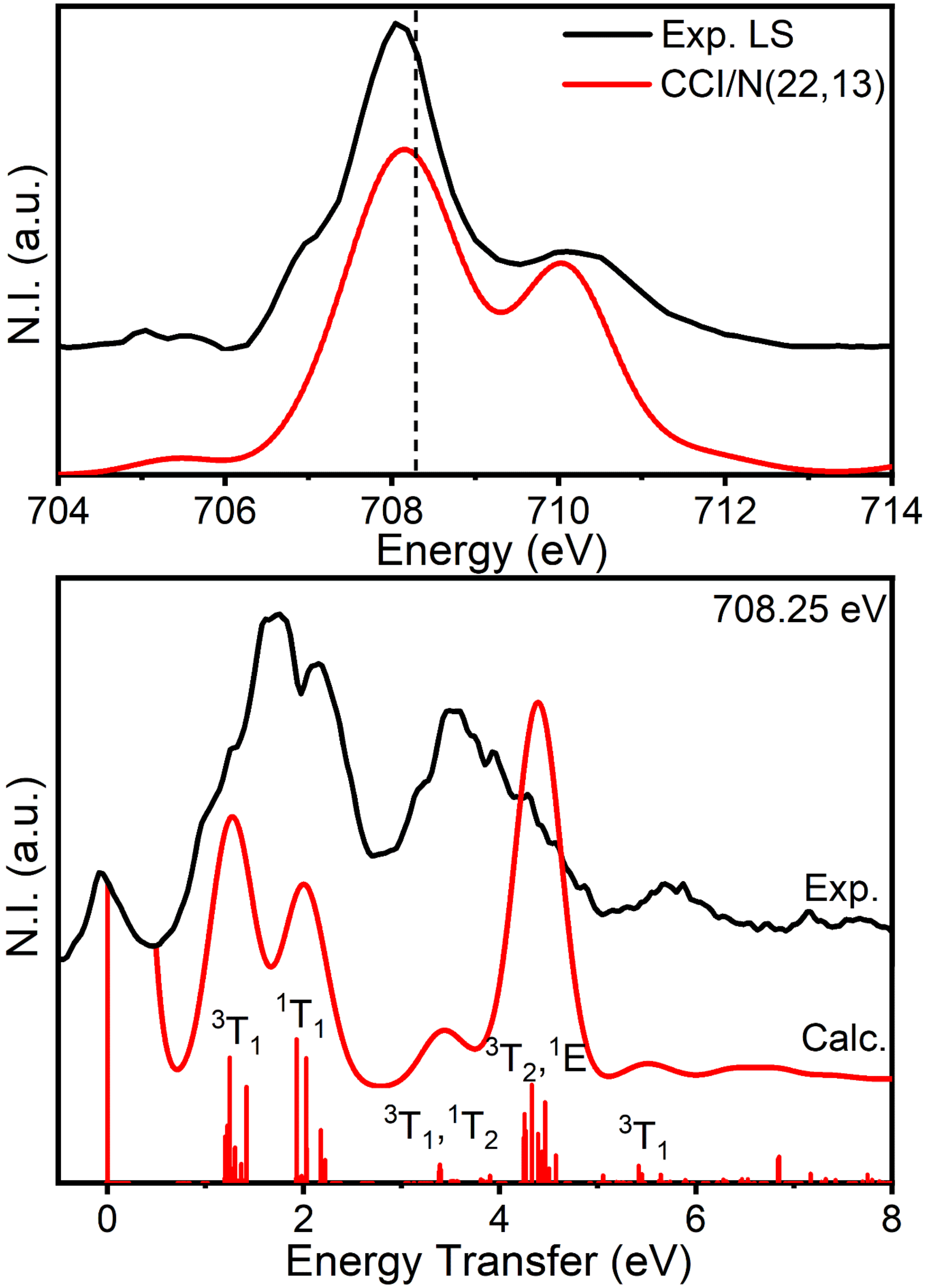

Figure S12. (top) Fe L_3-edge of LS **1** (black) compared with that calculated at the SA-CASCI/NEVPT2(22,13) level (red). An energy shift of -0.55 eV is applied to the calculated spectrum. A dashed vertical line indicates the incident energy used to collect the spectra in the bottom graph. (bottom) Experimental (black) vs. SA-CASCI/NEVPT2(22,13) calculated (red) Fe 2p3d RIXS at an incidence energy of 708.25 eV. The calculated Fe 2p3d RIXS represents the calculated spectrum at a 708.8 eV incident energy.

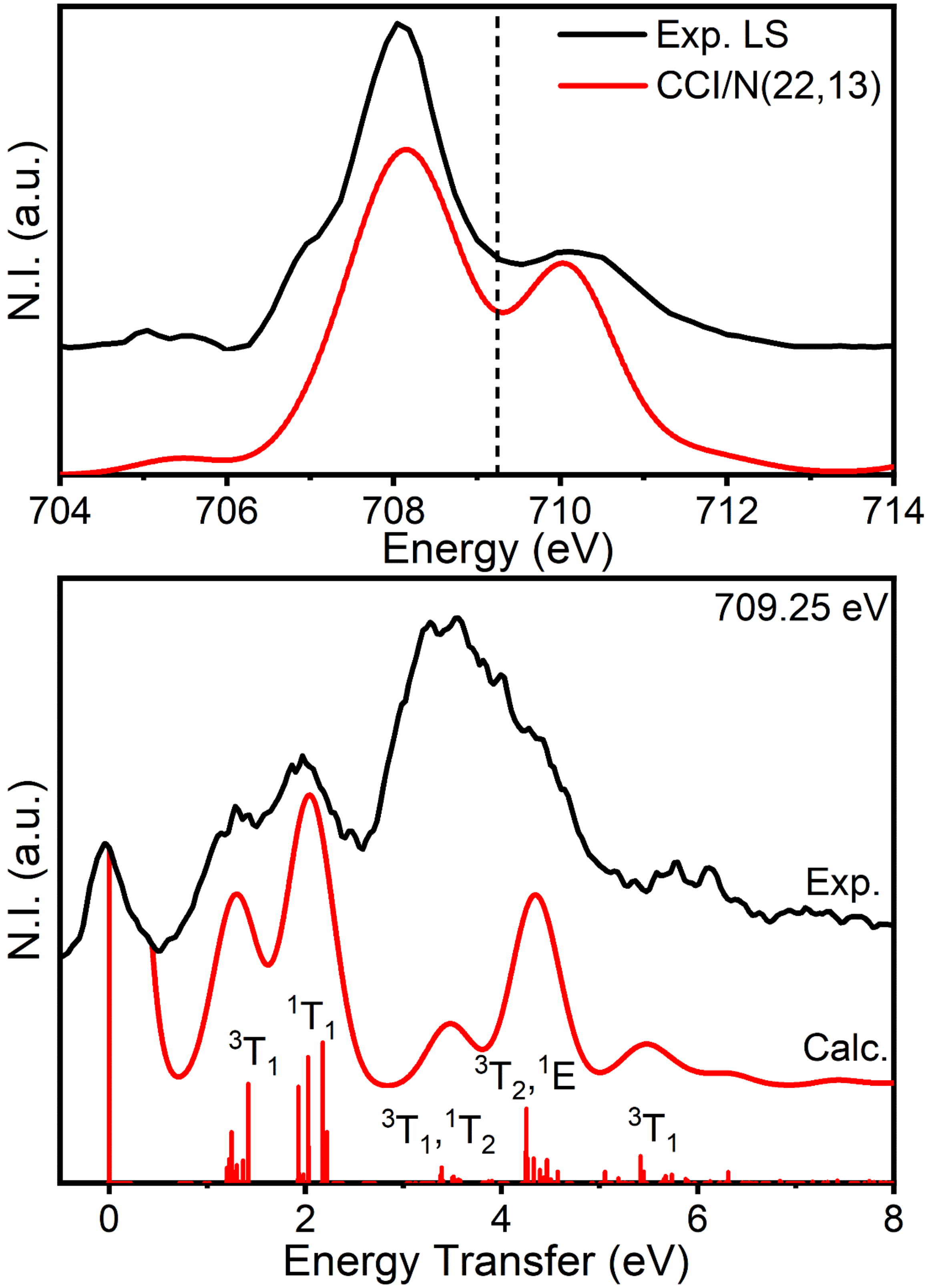

Figure S13. (top) Fe L_3-edge of LS **1** (black) compared with that calculated at the SA-CASCI/NEVPT2(22,13) level (red). An energy shift of -0.55 eV is applied to the calculated spectrum. A dashed vertical line indicates the incident energy used to collect the spectra in the bottom graph. (bottom) Experimental (black) vs. SA-CASCI/NEVPT2(22,13) calculated (red) Fe 2p3d RIXS at an incidence energy of 709.25 eV. The calculated Fe 2p3d RIXS represents the calculated spectrum at a 709.8 eV incident energy.

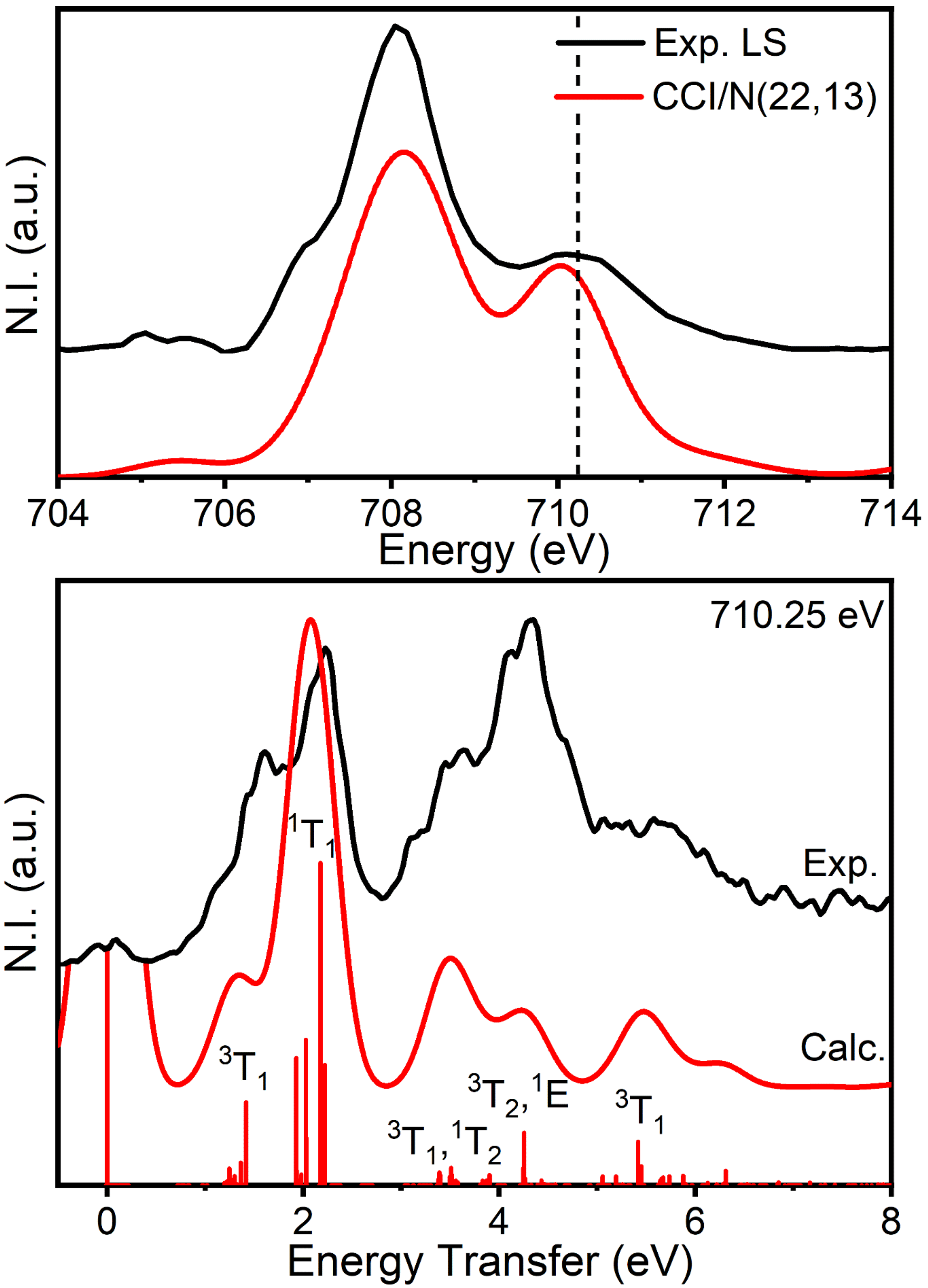

Figure S14. (top) Fe L_3-edge of LS **1** (black) compared with that calculated at the SA-CASCI/NEVPT2(22,13) level (red). An energy shift of -0.55 eV is applied to the calculated spectrum. A dashed vertical line indicates the incident energy used to collect the spectra in the bottom graph. (bottom) Experimental (black) vs. SA-CASCI/NEVPT2(22,13) calculated (red) Fe 2p3d RIXS at an incidence energy of 709.25 eV. The calculated Fe 2p3d RIXS represents the calculated spectrum at a 709.8 eV incident energy.

7. Band Deconvolution Analysis

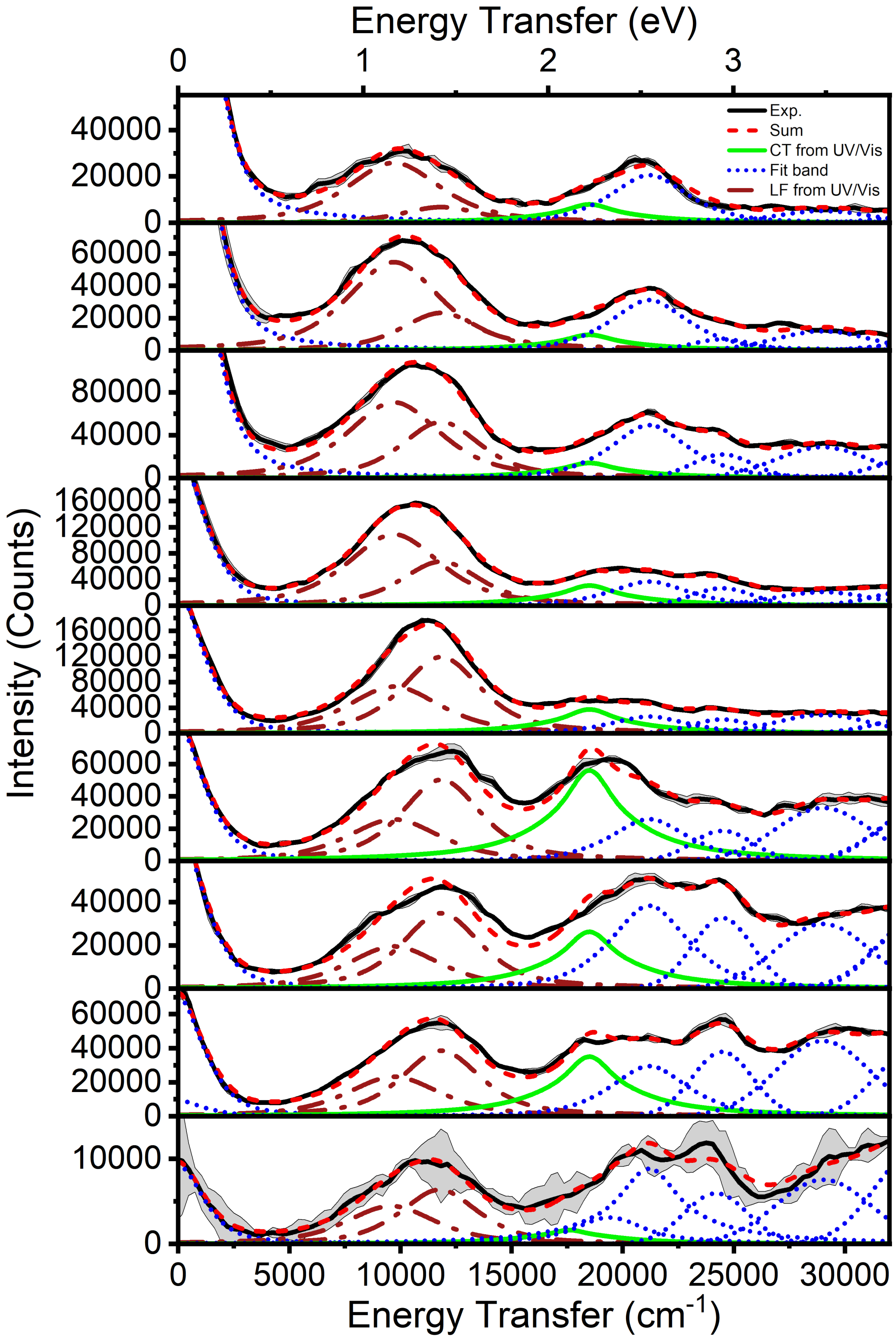

Figure S15. Pseudo-Voigtian band deconvolution analysis of the RIXS of **1** collected at 200 K. Experimental data are plotted in black, individual fit bands in blue (dotted), dark red (dash dot), and green (solid), and the sum of the fit in red (dashed). Incident energies used for the collection are indicated in the upper right corner of each individual spectra. Standard deviations (σ) for each spectrum are provided as a shaded gray area.

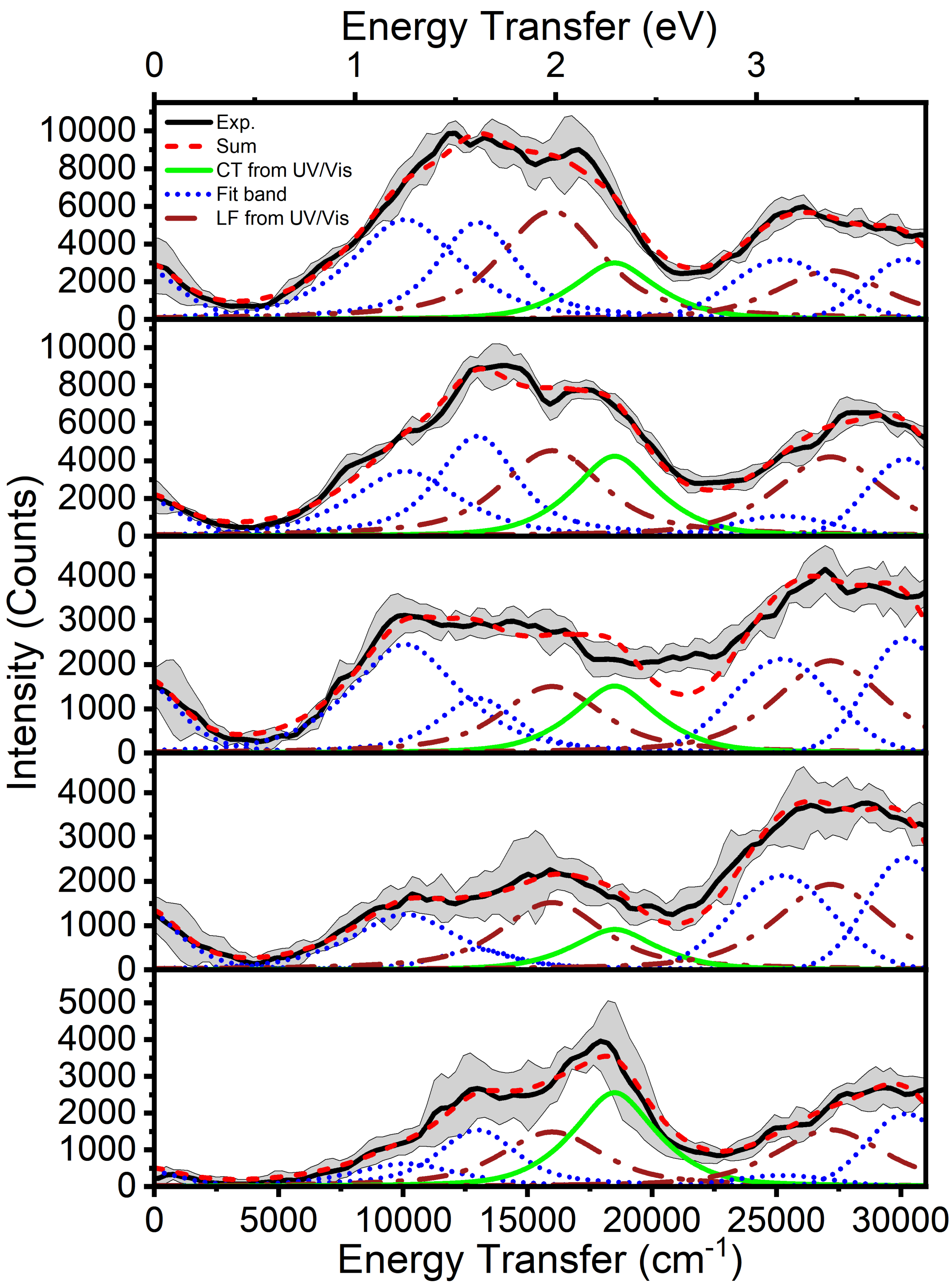

Figure S16. Pseudo-Voigtian band deconvolution analysis of the RIXS of **1** collected at 50 K. Experimental data are plotted in black, individual fit bands in blue (dotted), dark red (dash dot), and green (solid), and the sum of the fit in red (dashed). Incident energies used for the collection are indicated in the upper right corner of each individual spectra. Standard deviations (σ) for each spectrum are provided as a shaded gray area.

Table S1. Summary of SA-CASSCF/NEVPT2 calculated LF states of HS **1** below 40,000 cm^{-1} (~5 eV) averaged for an O_h ligand-field.

	Active Space			
	(6,5)		(16,10)	
State	eV	cm^{-1}	eV	cm^{-1}
1-^5T_2	0	0	0	0
1-^1A_1	1.26	10,150	1.24	9,970
1-^3T_1	1.30	10,460	1.27	10,280
1-^5E	1.43	11,550	1.38	11,150
1-^3T_2	1.91	15,400	1.88	15,140
1-^1T_1	2.47	19,955	2.46	19,850
2-^3T_1	2.50	20,145	2.48	20,360
2-^3T_2	2.70	21,780	2.69	22,110
1-^3E	2.86	23,050	2.81	22,810
3-^3T_1	2.96	23,840	2.96	24,060
1-^1T_2	3.49	28,170	3.42	27,590
3-^3T_2	3.50	28,225	3.48	28,050
2-^1A_1	3.70	29,830	3.58	28,900
1-^3A_2	3.64	29,335	3.61	29,120
1-^1E	3.78	30,510	3.80	30,650
2-^1T_2	3.95	31,860	3.93	31,720
3-^3T_1	3.97	31,990	3.91	31,510
3-^3E	4.05	32,695	3.99	32,220
4-^3T_2	4.28	34,490	4.23	34,150
1-^3A_1	4.40	35,495	4.35	35,060
2-^1T_1	4.42	35,630	4.37	35,250
1-^1A_2	4.57	36,865	4.53	36,510
4-^3T_1	4.84	39,030	4.74	38,210
3-^1T_2	4.95	39,940	4.87	39,300

Table S2. Summary of SA-CASSCF/NEVPT2 calculated LF states of LS **1** below 40,000 cm^{-1} (~5 eV), averaged for an O_h ligand-field.

	Active Space			
	(6,5)		(16,10)	
State	eV	cm^{-1}	eV	cm^{-1}
1-^1A_1	0	0	0	0
1-^5T_2	0.99	8,015	1.10	8,850
1-^3T_1	1.17	9,420	1.19	9,565
1-^3T_2	1.92	15,485	1.91	14,405
1-^1T_1	2.28	18,370	2.28	18,420
1-^3E	3.43	27,630	3.45	27,795
1-^5E	3.53	28,480	3.52	28,420
1-^1T_2	3.68	29,670	3.58	28,430
2-^3T_2	3.79	30,600	3.79	30,605
2-^3E	3.89	31,410	3.94	31,790
3-^3T_2	4.41	35,570	4.42	35,647
1-^1E	4.57	36,820	4.59	37,045
2-^1T_2	4.65	37,535	4.68	37,720
2-^1A_1	4.73	38,145	4.86	39,195

References

1. Moliner, N.; Salmon, L.; Capes, L.; Muñoz, M. C.; Létard, J.-F.; Bousseksou, A.; Tuchagues, J.-P.; McGarvey, J. J.; Dennis, A. C.; Castro, M.; Burriel, R.; Real, J. A., Thermal and Optical Switching of Molecular Spin States in the {[FeL[$H_2B(pz)_2]_2$} Spin-Crossover System (L = bpy, phen)†. *J. Phys. Chem. B* **2002,** *106*, 4276-4283.
2. Real, J. A.; Munoz, M. C.; Faus, J.; Solans, X., Spin Crossover in Novel Dihydrobis(1-pyrazolyl)borate [$H_2B(pz)_2$]-Containing Iron(II) Complexes. Synthesis, X-ray Structure, and Magnetic Properties of [FeL{$H_2B(pz)_2\}_2$] (L = 1,10-Phenanthroline and 2,2'-Bipyridine). *Inorg. Chem.* **1997,** *36*, 3008-3013.
